# Microcystin shapes the *Microcystis* phycosphere through community filtering and by influencing cross-feeding interactions

**DOI:** 10.1093/ismeco/ycae170

**Published:** 2024-12-24

**Authors:** Rebecca Große, Markus Heuser, Jonna E Teikari, Dinesh K Ramakrishnan, Ahmed Abdelfattah, Elke Dittmann

**Affiliations:** Department of Microbiology, Universität Potsdam, Institute of Biochemistry and Biology, 14476 Potsdam-Golm, Germany; Department of Microbiology, Universität Potsdam, Institute of Biochemistry and Biology, 14476 Potsdam-Golm, Germany; Department of Microbiology, Faculty of Agriculture and Forestry, University of Helsinki, Helsinki 00790, Finland; Institute for Atmospheric and Earth System Research, University of Helsinki, Helsinki 00790, Finland; Department of Agricultural Sciences, University of Helsinki, Falculty of Agriculture and Forestry, Helsinki 00790, Finland; Department for Microbiome Biotechnology, ATB Leibniz-Institute for Agriculture and Bioeconomy, 14469 Potsdam-Bornim, Germany; Department for Microbiome Biotechnology, ATB Leibniz-Institute for Agriculture and Bioeconomy, 14469 Potsdam-Bornim, Germany; Department of Microbiology, Universität Potsdam, Institute of Biochemistry and Biology, 14476 Potsdam-Golm, Germany

**Keywords:** phototroph-heterotroph interactions, cyanobacterial blooms, microcystin

## Abstract

The cyanobacterium *Microcystis* causes harmful algal blooms that pose a major threat to human health and ecosystem services, particularly due to the prevalence of the potent hepatotoxin microcystin (MC). With their pronounced EPS layer, *Microcystis* colonies also serve as a hub for heterotrophic phycosphere bacteria. Here, we tested the hypothesis that the genotypic plasticity in its ability to produce MC influences the composition and assembly of the *Microcystis* phycosphere microbiome. In an analysis of individual colonies of a natural *Microcystis* bloom, we observed a significantly reduced richness of the community in the presence of MC biosynthesis genes. A subsequent synthetic community experiment with 21 heterotrophic bacterial strains in co-cultivation with either the wild-type strain *Microcystis aeruginosa* PCC 7806 or the MC-free mutant Δ*mcyB* revealed not only a tug-of-war between phototrophic and heterotrophic bacteria, but also a reciprocal dominance of two isolates of the genus *Sphingomonas* and *Flavobacterium*. In contrast, an *Agrobacterium* isolate thrived equally well in both consortia. In substrate utilization tests, *Sphingomonas* showed the strongest dependence on *Microcystis* exudates with a clear preference for the wild-type strain. Genome sequencing revealed a high potential for complementary cross-feeding, particularly for the *Agrobacterium* and *Sphingomonas* isolates but no potential for MC degradation. We postulate that strain-specific functional traits, such as the ability to perform glycolate oxidation, play a crucial role in the cross-feeding interactions, and that MC is one of the determining factors in the *Microcystis* phycosphere due to its interference with inorganic carbon metabolism.

## Introduction

Massive growth events (blooms) of cyanobacteria are a seasonally recurring problem that poses a threat to human health, water quality and ecosystem services [1]. The spread of these blooms is increasingly accelerated due to rising eutrophication, but also through higher temperatures across the globe, and longer stratification periods as a result of climate change [2–4]. A widespread cyanobacterial genus that tends to form harmful algal blooms is the unicellular genus *Microcystis* which is a predominant producer of the potent hepatotoxin microcystin (MC) [5]. *Microcystis* sp*.* forms macroscopically visible colonies that are surrounded by a distinct mucus layer, which serves as a nutrient-rich habitat for the heterotrophic microbiome (phycosphere) [6]. *Microcystis* and its heterotrophic interactome are increasingly regarded as holobiont [6], with the heterotrophic bacteria benefiting from the dissolved organic carbon provided by the cyanobacteria and the cyanobacteria presumably taking advantage of nutrient recycling, CO_2_ production and the reduction of reactive oxygen species by their microbiome [7–11]. Cultivation-dependent studies have shown that a significant number of the associated heterotrophic bacteria have a growth-promoting effect on *Microcystis* strains [12]. Extensive studies of the *Microcystis* phycosphere have provided evidence of a high specificity of interactions compared to the surrounding planktonic bacterial community [7]. At the same time, however, variability and succession of the microbial community are also observed, especially in different bloom stages during the season [13].

The great environmental success of *Microcystis* is increasingly associated with the genotypic and phenotypic plasticity of the genus [14, 15]. A functional trait that shows great variability among *Microcystis* strains is the inorganic carbon adaptation. Different *Microcystis* strains encode different sets of bicarbonate uptake transporters, resulting in major differences among individual strains in their adaptation to low or high availability of inorganic carbon [16, 17]. Further, a large part of the flexible genome portions is dedicated to the production of different secondary metabolites, including MC [14, 18]. Notably, the genotypic and chemotypic plasticity of *Microcystis* is reflected in the associated microbiome. A recent study was able to provide clear indications of a co-phylogeny through single colony sequencing [19]. However, little is known about which flexible *Microcystis* traits have the greatest impact on the specificity of the interactions. There are already indications that the ability to produce MC has an influence on heterotrophic partners. For example, one study showed a positive correlation between the ability to produce toxins and the occurrence of α-proteobacteria of the genus *Phenylobacterium* in Lake Taihu in China [20].

There is growing evidence that the ecological role of secondary metabolites extends well beyond their potential role in defense and includes influences on microbial growth, biofilm formation and community behavior [21, 22]. In the case of MC, the defensive function is largely limited to eukaryotic organisms. A role for MC in microbe-microbe interactions is therefore currently rather discussed in connection with the specific degradation of MC and its use as a carbon source [23]. However, there is extensive evidence that the ability to produce MC has an impact on functional traits that can potentially influence microbial interactions. For example, the phenotypic, proteomic and metabolomic comparison of the toxic strain *M. aeruginosa* PCC 7806 and the Δ*mcyB* mutant showed that (i) the loss of MC affects surface components such as the lectin Mvn and the filamentous glycoprotein MrpC and concomitantly the aggregation tendency of the bacteria [24, 25]; (ii) the loss of MC leads to a reprogramming of the carbon metabolism, especially under high light conditions, which also influences the accumulation of extracellular dissolved organic carbon in the form of glycolate [26]; (iii) the loss and also the addition of MC affects the subcellular localization of the CO_2_-fixing enzyme RubisCO, which could possibly promote direct CO_2_ assimilation from the heterotrophic bacteria [27, 28].

In the present study, we aimed to systematically investigate the influence of MC production on the composition of the *Microcystis* microbiome. To this end, we first performed single colony analyses of MC-producing and non-producing field colonies employing PCR-based discrimination and 16S-rRNA gene amplicon sequencing strategies. We then established and analyzed a synthetic community covering a broad spectrum of phycosphere bacteria. We were able to show a correlation between MC production and the composition of the microbiome in both field and laboratory samples and further provide evidence that the exudates of cyanobacteria have a discriminating effect on the growth of individual heterotrophic species. Our study supports the hypothesis that MC is one of the flexible traits that influences the specificity of interactions in *Microcystis* holobionts.

## Materials and methods

### Isolation of single colonies and heterotrophic bacterial isolates

Single *Microcystis* colonies were sampled and isolated during a bloom event on July 14th, 2021, at three locations along the lake Wublitz (Site 1: 52.417028741058964, 12.935628335139363; Site 2: 52.428188547155465, 12.936549043106284; Site 3: 52.425179744399784, 12.93285196111643). Detailed information on the water body can be obtained from the website https://undine.bafg.de/elbe/guetemessstellen/elbe_mst_potsdam_humboldtbr.html provided by the Brandenburg state environmental agency. A detailed description on the isolation procedure is provided in the Supplementary Method S1.

Heterotrophic bacteria were isolated from the same water samples and non-axenic cyanobacterial lab strains. Briefly, one drop of lake water containing 1–2 *Microcystis* colonies was streaked on CYA Agar [12] or R2A agar plates (Carl Roth, Karlsruhe, Germany) and incubated at 25°C in the dark. Colonies were repeatedly picked and streaked until single colonies with unique bacterial species could be obtained, based on visual inspection of colony morphology and color. From non-axenic cyanobacterial lab strains, 10 μl were streaked on R2A agar plates and isolated identically to the lake water samples.

### Heterotrophic bacterial strain identification

Heterotrophic bacterial isolates were identified using Sanger sequencing of the 16S-rRNA gene amplicons. The MEGA X software (version 11.0.13) was used to create a phylogenetic tree. A detailed description of PCR and sequence data processing is provided in the supplementary Method S2.

### Co-cultivation experiment

Pre-cultures of cyanobacterial strains *Microcystis aeruginosa* PCC 7806 wild type (WT) and MC-LR deficient Δ*mcyB* mutant were grown in BG11 medium [29] on the benchtop to adapt to a day–night cycle and a temperature of 25°C for at least one week. Heterotrophic bacterial cultures were grown on agar plates. Further descriptions are provided in the supplementary Method S3. For the co-cultivation experiment, synthetic communities were composed of *M. aeruginosa* PCC 7806 WT and Δ*mcyB* mutant together with a consortium of 21 heterotrophic bacterial isolates (Table S3). For the experiment set up, triplicates were prepared as follows: Pre-cultures of WT and Δ*mcyB* mutant were centrifuged (4700*g, 10 min, RT), washed and resuspended in fresh nitrogen-containing BG11 medium without chloramphenicol to a final OD_720_ of 0.2, which corresponded to a cell number of 6*10^6^ cells/ml. Pre-cultures of heterotrophs were grown in their respective liquid media for 2–4 days, washed and resuspended in fresh BG11 medium. Bacterial cell suspensions were diluted so that the final amount of each heterotroph isolate should have a cell number of 1/21th of the *Microcystis* cell number (~3*10^5^). The experiment was run over a time course of 28 days, and samples for DNA extraction (10 ml) were taken weekly. For the incubation, the MultiCultivator OD-1000 (Photon Systems Instruments, Drásov, Czech Republic) was used. A constant temperature of 25°C was maintained for the entire experiment and the following day–night light regime was applied: 15.5 h light (55 μmol photons m^−2^ s^−1^), 30 min linear light reduction phase to 0 μmol photons m^−2^ s^−1^, 7.5 h of darkness (0 μmol photons m^−2^ s^−1^) followed by a linear light increasement phase from 0 to 55 μmol photons m^−2^ s^−1^ in 30 min. OD_720_ was measured automatically every 5 min. Axenicity of cyanobacterial monocultures was confirmed by 16S-rRNA gene amplicon sequencing*.* To confirm the presence of intra- and extracellular MC, a small-scale co-cultivation experiment was performed consisting of *Sphingomonas*, *Agrobacterium,* and *Flavobacterium* together with *M. aeruginosa* PCC 7806 WT and Δ*mcyB* mutant and analyzed by HPLC and LC–MS methods (detailed descriptions of the set-up are provided in the supplementary Method S4, Table S5 and Table S6).

### DNA extraction

DNA extraction from the single colonies was done using the ChargeSwitch gDNA Mini Bacteria Kit (Thermo Fisher Scientific, Waltham, MA, USA), as previously described by Pérez-Carrascal, et al. [19]. DNA extraction from synthetic communities was performed using a modified protocol for the DNeasy Blood&Tissue kit (Qiagen, Hilden, Germany). Detailed descriptions are provided in the supplementary Method S5.

### Chemotype identification of single colonies

The chemotype of 29 single *Microcystis* colonies was classified into 12 MC-producing (*mcyA(+)*) and 17 non-producing (*mcyA(−)*) colonies based on the presence/absence of an NRPS gene from the MC biosynthetic gene cluster as described in supplementary Method S6.

### 16S-rRNA gene amplicon sequencing and microbial community analysis

Microbiome analysis was performed based on the sequencing of the 16S-rRNA V3-V4 gene region. Rarefaction curves are shown in Fig. S1. A detailed description of sequencing methods and data processing is given in Supplementary Method S7.

### Metabolic profiling with EcoPlates

Utilization of specific substrates by the three heterotrophic bacterial strains *Agrobacterium, Flavobacterium, Sphingomonas* was done using EcoPlate (Biolog Inc., Hayward, CA, USA). A detailed description of the experimental set-up is provided in the supplementary Method S8.

### Genome sequencing and annotation

Genome sequencing and annotation of the three strains *Agrobacterium* sp UP1*, Flavobacterium* sp*.* UP2*,* and *Sphingomonas* sp*.* UP3 was performed according to supplementary Method S9*.*

## Results

*Richness and community composition differ in MC+ and MC*- Microcystis *phycospheres.*

**Figure 1:**
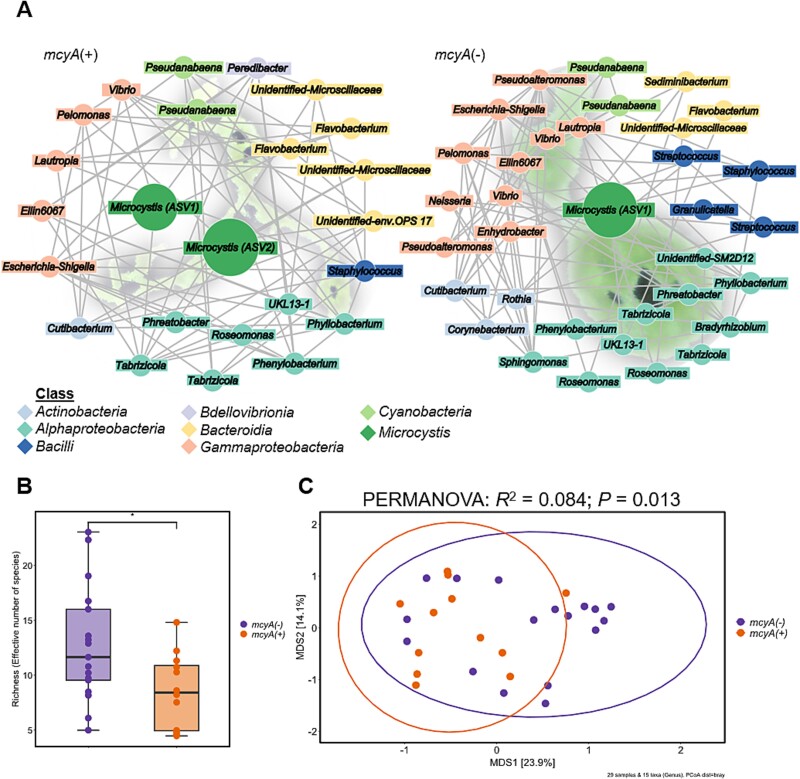
**Analysis of naturally occurring microbiomes of single *Microcystis* colonies.** Microbiome data of single colonies were grouped based on the presence (*mcyA(+)*) or absence (*mcyA(−)*) of the microcystin-producing gene *mcyA*. (A) Co-occurrence networks of *mcyA(+)* and *mcyA(−)* bacterial communities on ASV level. Nodes were colored according to the respective taxonomic class, except *Microcystis* was colored according to genus. Node size reflects relative abundance. The backgrounds depict example *Microcystis* colonies. Edges connect nodes that share co-presence with more than one other node. (B) Richness of *mcyA(+)* and *mcyA(−)* single colony communities. Each dot represents the richness of a single colony in the respective group (*n*(*mcyA(−)*) = 17; *n*(*mcyA(+)*) = 12). Box plots show the median (horizontal line), the lower and upper bounds of each box plot indicate the first and third quartiles and whiskers above and below the box plot show 1.5 times the interquartile range. The asterisk represents significant difference (*P* < .05, Wilcoxon rank sum exact test & linear regression modelling). (C) Principal Co-ordinates analysis (PCoA) plot on Bray–Curtis dissimilarities of single colony community composition on genus level. The ellipses represent 95% confidence intervals. Color of points, boxes and ellipses correspond to samples with presence and absence of *mcyA*-gene.

To analyze a possible correlation of MC and *Microcystis* phycosphere microbiomes, we isolated the DNA of 29 single colonies from an early-stage *Microcystis* bloom in lake Wublitz near Potsdam (Fig. S2)*.* Colonies were sampled on the same day to minimize the influence of seasonality, abiotic environmental factors, and the surrounding free-living heterotrophic community. PCR analysis of the MC biosynthesis gene *mcyA* was used to discriminate individual colonies into MC+ (12; *mcyA(+)*) and MC- genotypes (17; *mcyA(−)*) (Fig. 1A, Figs. S3 and S4). We assume that in most of colonies, the presence of *mcyA* correlates with MC-production [30], but we cannot rule out the possibility that some colonies do not produce MC despite the presence of the *mcyA* gene, as recently was shown [31]. Next, using the same DNA material, the microbiomes of MC+ and MC- colonies were studied by sequencing 16S-rRNA V3-V4 gene amplicons. Following taxonomy assignment, individual colonies could be assigned to three different *Microcystis* ASVs (> 1000 reads). Specifically, a single dominant *Microcystis* ASV was detected in 80% of the analyzed colonies (10 colonies belonged to ASV1, 12 colonies to ASV2, and one colony to ASV3, Table S1), whereas a mixture of two different *Microcystis* ASVs was observed in 20% of colonies. A similar observation was recently made in a single colony study of Lake Erie *Microcystis* blooms and discussed as a result of two divergent 16S-rRNA gene copies in single *Microcystis* genomes [32]. Based on these findings, we assume that the individual colonies isolated in this study were predominantly clonal isolates. Yet, we cannot exclude the possibility that some of the colonies contained two clonal strains. We did not observe a correlation between ASV type and the presence or absence of MC. Next, we analyzed the non-*Microcystis* ASVs in single colonies. Similar to previous studies, we did not observe a ubiquitously present core microbiome. However, certain taxa showed comparatively high prevalence. Among all colonies, the predominant heterotrophic taxa detected on genus level were *Roseomonas* and *Microscillaceae Family* (76%), *Vibrio* (72%), *Pelomonas, Tabrizicola* and *Cutibacterium* (69%), and *Phenylobacterium* and *Flavobacterium* (62%) (Figs. 1A, S4, and Table S2). To evaluate possible differences between MC+ and MC- microbiomes we compared co-presence networks for the two subgroups. Both networks were found to be principally similar, with a total of 20 nodes assigned to genera that are shared between the two types (Figs. 1A andS4). However, there were also some noticeable differences. Overall, the total number of non-*Microcystis* nodes was lower in the MC+ type (24 nodes) than in the MC- type (34 nodes). Based on this observation, we set out to estimate the richness on the genus level for the two groups (Fig. 1B). To focus on the effect on the heterotrophic microbiome, cyanobacterial ASVs were excluded prior to this analysis. As already indicated by the findings of the co-presence network, the presence of the *mcyA* gene was indicative of a significantly lower richness (*P* < .05, Wilcoxon rank sum test, LRM). This suggests that MC-producers establish a less diverse and thus potentially higher specialized microbiome. In order to test the similarity of the phycosphere microbiomes of MC+ and MC- colonies, we used PCoA ordination to visualize Bray-Curtis dissimilarities together with permutational multivariate analysis of variance (PERMANOVA). This demonstrated a significant difference between the chemotypes (PERMANOVA: *R*^2^ = 0.084, *P* = .013), although it accounted only for 8.4% of the variation, suggesting that the ability to produce MC is a minor contributor to the overall variation (Fig. 1C). To identify differentially abundant taxa in both groups, we used LEfSe analysis [33]. Three taxa were scored in the MC+ colonies: *Tabrizicola*, *Phenylobacterium,* and a *Microscillaceae* family member. In the MC- group, two taxa were scored: *Cutibacterium* and *Streptococcus* (Fig. S5). Taken together, these findings suggest that the genetic predisposition for MC production might support *Microcystis* in their ability to filter heterotrophic bacterial partners in their phycosphere.

### Temporal dynamics of synthetic communities in co-culture with an MC+ and an MC- laboratory strain

**Figure 2:**
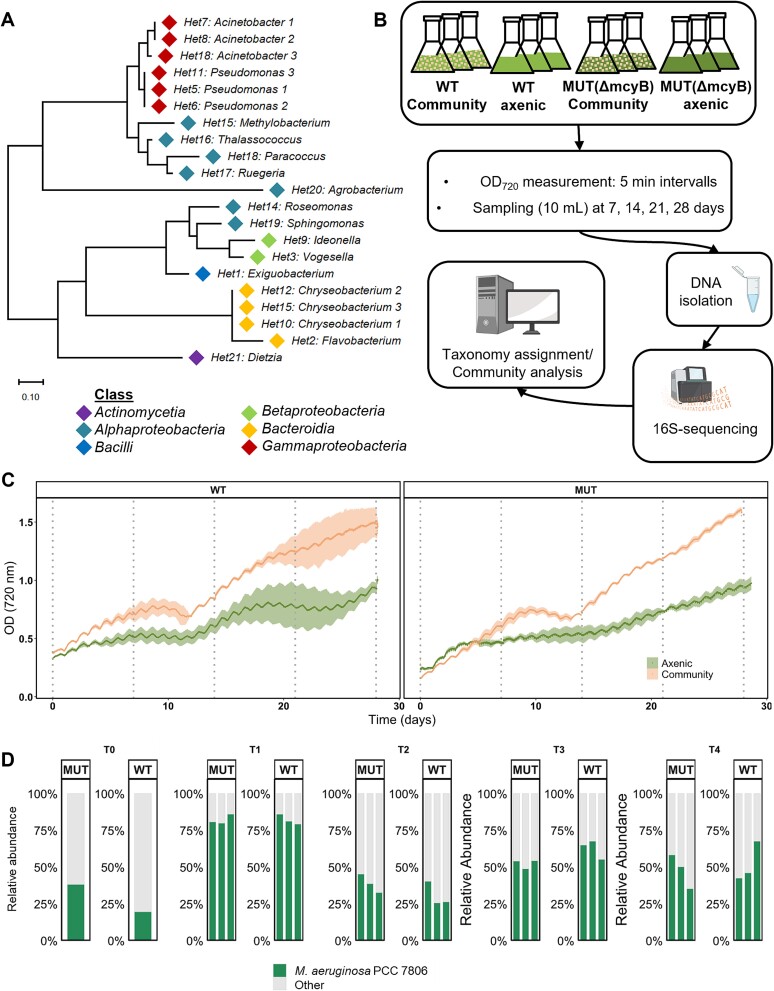
**Characteristics of the synthetic community experiment realized through co-cultivation of the MC-producing *Microcystis aeruginosa* PCC 7806 WT or the non-producing Δ*mcyB* mutant together with 21 bacterial isolates.** (A) Phylogenetic tree of 21 bacterial isolates used to assemble the heterotrophic bacterial consortium with identified genus label and color-coded by class. Numbers behind the taxa label indicate separate isolates of the same genus. Detailed descriptions about isolates are shown in Table S3. (B) Illustration of the experimental workflow. Four different conditions were tested: Axenic *M. aeruginosa* PCC 7806 (WT axenic), axenic non-producing mutant (Δ*mcyB* axenic), *M. aeruginosa* PCC 7806 WT in co-cultivation with the synthetic consortium (WT community) and the non-producing mutant in co-cultivation with the synthetic consortium (Δ*mcyB* community). Three biological replicates were established for each condition (*n* = 3). The cultures were subjected to a day–night cycle of 15 h of constant daylight (55 μmol/m^2^*s) and 7 h constant darkness (0 μmol/m^2^*s). Transitions between day and night phases were implemented with linear change of light intensity and the respective target light intensity was reached after 30 min. Automated measurement of optical density (OD) was done at 720 nm throughout the whole course of the experiment every 5 min. Samples were taken weekly over a period of 28 days followed by 16S-rRNA gene amplicon sequencing and analysis. Parts of the illustration were created with biorender.com. (C) Development of OD_720_ of *M. aeruginosa* PCC 7806 WT and Δ*mcyB* mutant over time, colored according to experimental condition. Deep-colored lines represent the mean of *n* = 3 (axenic condition) and *n* = 2 (community condition) replicates. Vertical dotted lines indicate sampling time points. Standard deviation is shown as pale ribbons. (D) Relative abundance of *Microcystis* at each sampling timepoint in WT and Δ*mcyB* mutant co-cultivation. Each bar represents a replicate.

To further test the hypothesis that the presence of MC has an influence on the *Microcystis* phycosphere composition we designed a 28-day synthetic community experiment with the MC-producing laboratory strain *M. aeruginosa* PCC 7806 (WT) and its MC-deficient Δ*mcyB* mutant together with a defined consortium of 21 heterotrophic bacterial strains (Figs. 2A and B). To establish a synthetic consortium, 13 different heterotrophic bacteria were isolated from a *Microcystis* bloom sample of lake Wublitz. The isolates included both representatives that are frequently associated with *Microcystis* colonies (*Flavobacterium* [8, 12, 13, 34, 35] and *Pseudomonas* [8, 12, 23, 36, 37]) and representatives that were only occasionally associated with *Microcystis* (*Acinetobacter* [10, 11], *Vogesella* [12, 38], *Exiguobacterium* [12, 38, 39] and *Chryseobacterium* [12, 40]). To better represent the natural diversity of *Microcystis* phycosphere microbiomes, we further included eight strains obtained from other phycosphere microbiomes sampled at the University of Jena, Germany or isolated as contaminants in cyanobacterial laboratory cultures (Table S3). These bacteria included strains of the taxa which are often co-occurring with *Microcystis* (*Sphingomonas* [8, 12, 36, 41, 42], *Roseomonas* [13, 32, 37], and *Paracoccus* [12, 43]), but also *Agrobacterium*, which has incidentally been described as a predominant *Microcystis* partner [11, 39, 44]. Sequences of strains isolated from cyanobacterial laboratory cultures (*Sphingomonas*, *Agrobacterium*) were compared against sequence read archives (SRA) from a *Microcystis* environmental metagenomic study (PRJNA507251 [19]) where partial V3-V4 sequence regions matched with maximal sequence identity of 100%.

Finally, we added *Methylobacterium*, *Thalassococcus*, *Ruegeria*, *Ideonella,* and *Dietzia* to cover a greater diversity of taxa at the class level, including *Alphaproteobacteria*, *Betaproteobacteria*, *Gammaproteobacteria*, *Bacteroidia*, *Actinomycetia*, and *Bacilli* (Fig. 2A and Table S3). During the course of the experiment, bacterial cells remained in suspension and no signs of flocculation or colony formation were observed (Fig. S6A and B). Monitoring of the OD_720_ showed that in both cases (WT and Δ*mcyB* mutant), the community condition ultimately reached higher values than the monocultures (Fig. 2C). Measurements of OD of heterotrophic bacteria alone also displayed absorbance at 720 nm, suggesting that the OD_720_ in the community is a mixed value of cyanobacterial and heterotrophic bacterial absorbance. However, higher OD_720_ values in the community might indicate that the presence of heterotrophic bacteria promoted an overall increased growth rate of *Microcystis* compared to the monoculture. A mutual influence on the growth dynamics of phototrophs and heterotrophs is indicated by an interesting phenomenon in the community growth curves (Fig. 2C): after the initial exponential phase (Day 0–9), the cultures transitioned into an intermittent stationary phase, where OD_720_ values visibly receded (Day 10–12). Remarkably, the cultures advanced into a second exponential growth phase that lasted until the end of the experiment (Day 28).

To estimate the relative abundance of *Microcystis* at different time points we further used 16S-rRNA gene amplicon sequencing. Taxonomic differentiation of *Microcystis* and non-*Microcystis* amplicons revealed pronounced temporal dynamics in the relative abundance of *Microcystis* during different stages of the co-cultivation experiment. At the beginning of the experiment the initial relative abundance of *Microcystis* was 19,5% and 38,1% in the WT and the Δ*mcyB* mutant strain, respectively (T0, Fig. 2D, Table S4). Maximal cyanobacterial relative abundance was observed in both consortia after one week (T1, Fig. 2D, Table S4) with 82,0% ± 3,4% in the WT and 82,1% ± 3,3% in the Δ*mcyB* mutant condition, followed by a steep decline after two weeks (WT 30,7% ± 8,3%; Δ*mcyB* mutant 38,7% ± 6,3%). This decline occurred simultaneously with the intermittent stationary phase in the growth curves (Fig. 2C), suggesting a potential correlation. From week three onwards, *Microcystis* relative abundance slightly increased and stabilized, with a final relative abundance of 52% ± 13,6% in the WT and 47,9% ± 11,6% in the Δ*mcyB* mutant, respectively (T4, Fig. 2D, Table S4).

**Figure 3:**
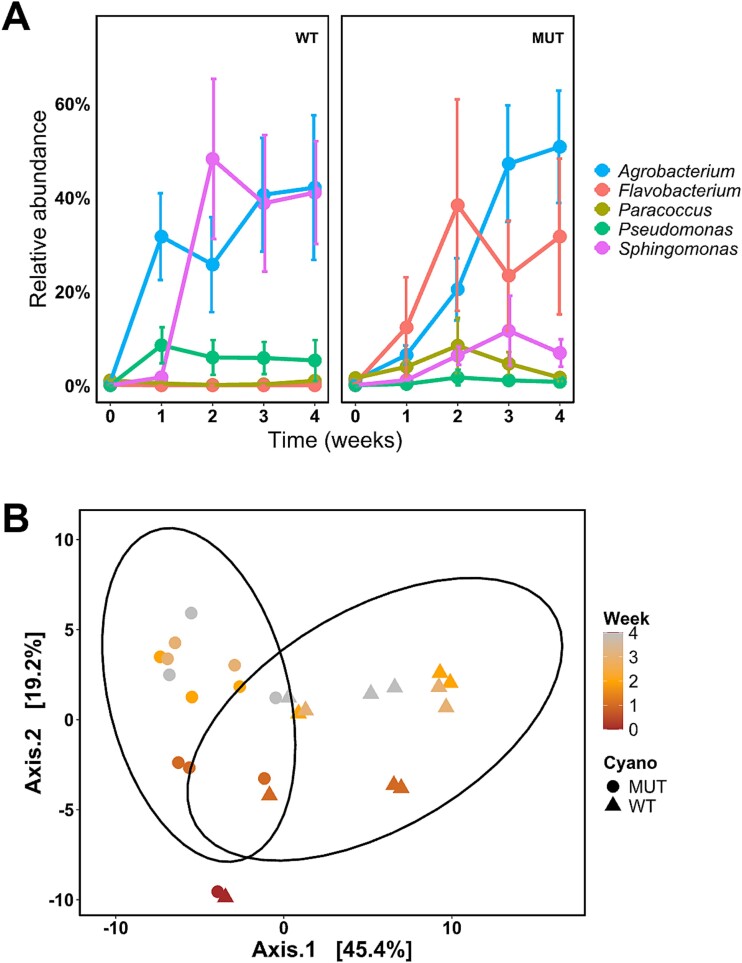
**Temporal dynamics of the synthetic community experiment.** (A) Longitudinal trajectories of significantly differential abundant taxa in co-cultivation experiments with *M. aeruginosa* PCC 7806 and Δ*mcyB* mutant shown as relative abundances (LEfSe analysis: *P* < .05 (Kruskal–Wallis-test), logLDA cutoff = 3, CSS normalization). Individual taxa are shown in different colors. Filled circles represent mean relative abundance and error bars represent standard deviation (*n* = 3). (B) Principal components analysis plot of sample composition on genus level. The first two dimensions are shown. Abundance data were transformed using the centered-log-ratio method. A pseudo count of half of the minimal relative abundance was added to exact zero relative abundance entries in the ASV table. Data points represent samples that are categorized by shape (WT = triangular, MUT (Δ*mcyB*) = circular), and by time (color gradient). The ellipses represent 95% confidence interval.

Throughout the co-cultivation experiments, the abundance of individual heterotrophic bacteria changed considerably. Although we aimed for similar proportions for all 21 heterotrophs in the inoculum, the genera *Vogesella* (68%) *Chryseobacterium* (19,1%), and *Methylobacterium* (7,8%) were in fact overrepresented at T0 (Fig. S7). However, they became less abundant over time and other genera became predominant (Fig. 3A, Fig. S7). The relative abundance ratios between cyanobacteria and heterotrophs remained stable after two weeks of cultivation, especially after T3 (Fig. 2D, Fig. S7). This could indicate that both cyanobacteria and heterotrophic bacteria reproduced in a constant and perhaps even synchronized manner during that period.

Temporal dynamics in the consortium composition were evaluated by Principal Components Analysis (Fig. 3B). The initially high community similarity between both WT and Δ*mcyB* seemed to develop into different directions already after one week. Maximum distance from the initial consortium and between the two genotypes was reached after two weeks and remained stable towards the end of the experiment (Fig. 3B).

Significantly differentially abundant heterotrophs were identified in each condition using LEfSe analysis (Fig. S8A and B). Longitudinal trajectories of the scored bacteria are shown in Fig. 3A. Reciprocal abundance development was observed for the *Sphingomonas* and the *Flavobacterium* strains, which showed dynamic growth exclusively in the WT and Δ*mcyB* mutant consortium, respectively (Fig. 3A). Besides, the *Agrobacterium* strain showed strong growth in both consortia regardless of the presence of MC.

### Metabolic profile and motility characteristics differ between differential abundant heterotrophs

**Figure 4:**
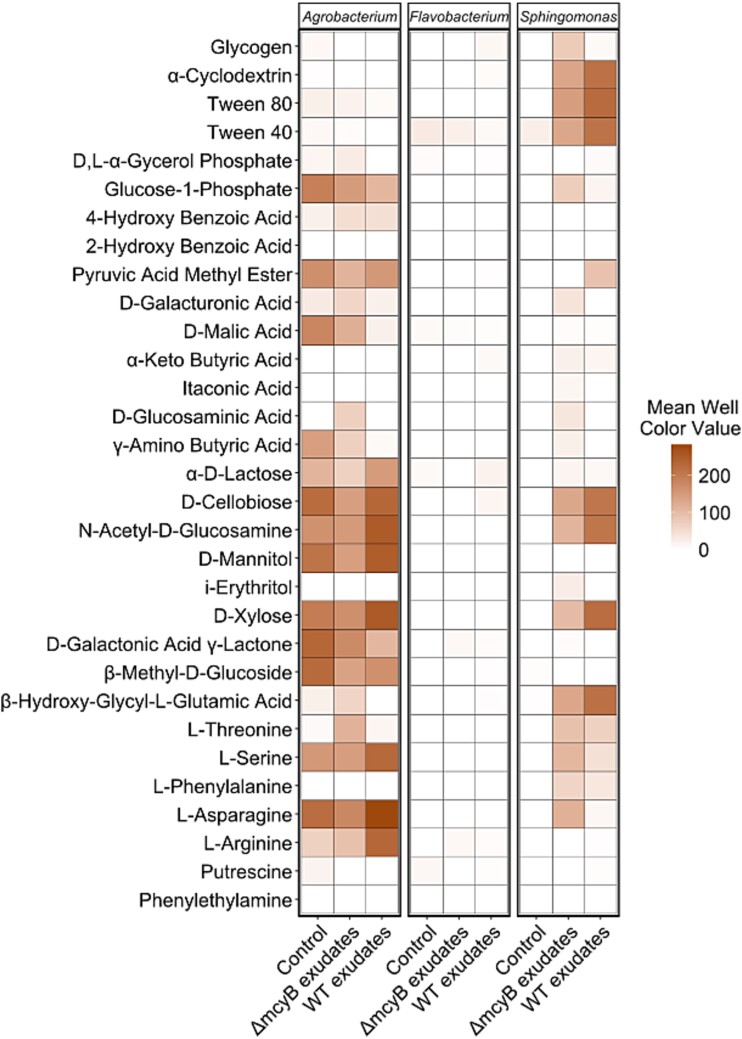
**Substrate utilization analysis using EcoPlate™ with and without supplementation of *Microcystis* exudates.** The heatmap shows mean well color values of biological replicates (*n* = 3) at the timepoint t = 100 h. Higher well color indicates higher substrate specific metabolic activity of the respective bacterium (*Agrobacterium* sp. UP1*, Flavobacterium* sp. UP2*, Sphingomonas* sp. UP3). Substrate specific metabolic activity was tested in control condition (0.9%-NaCl solution) or in presence of *M. aeruginosa* PCC 7806 WT or Δ*mcyB* mutant exudates. See Figs. S9–S11 for full 200 h time course for individual strains and substrates.

**Figure 5:**
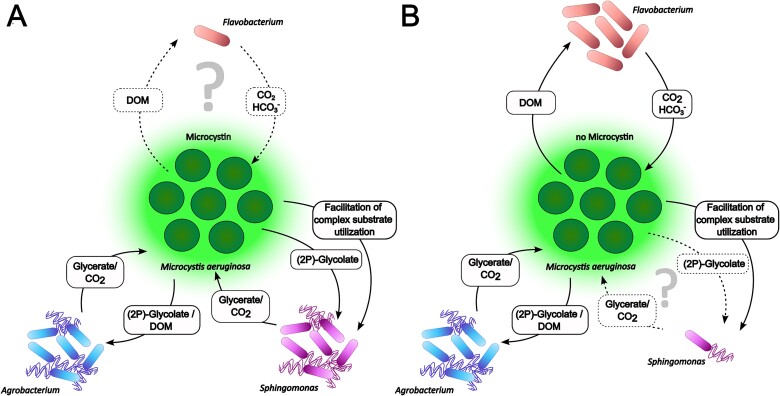
**Putative *Microcystis*-heterotroph interactions under MC+ or MC- conditions.** Schematic representation of the relationship between *Microcystis* and heterotrophic bacteria summarizing the findings of the synthetic community experiment, metabolic profiling, and KEGG pathway analysis based on genome sequencing. Number of heterotrophs in each condition reflects the detected relative abundances in the co-cultivation experiment (Fig. 4A). Putative interaction pathways indicated by arrows are based on the findings of metabolic profiling and KEGG pathway analysis of the respective genome. Motility of the heterotrophic bacteria is indicated by flagella. (A) In the presence of MC *Agrobacterium* and *Sphingomonas* reach high relative abundances, whereas *Flavobacterium* is low abundant. Suggested pathways of *Sphingomonas* and *Agrobacterium* are predominant. (B) In the absence of MC *Agrobacterium* and *Flavobacterium* reach high relative abundances, whereas *Sphingomonas* is low abundant. Suggested pathways of *Agrobacterium* and *Flavobacterium* are predominant.

To better understand the influence of MC on the interaction between cyanobacteria and heterotrophs, we subjected three selected strains (*Flavobacterium*, *Sphingomonas,* and *Agrobacterium*) to a metabolic profiling using EcoPlates™. To capture the influence of *Microcystis* exudates on strain specific growth and substrate utilization, we used sterile filtered *Microcystis* WT and Δ*mcyB* mutant culture exudates as liquid media for the incubation. *Microcystis* WT exudates contained extracellular MC whereas Δ*mcyB* mutant exudates were MC-free. Only *Agrobacterium* was able to convert substrates without the addition of *Microcystis* exudates, showing broad substrate utilization capabilities including for carbohydrates (e.g. D-cellobiose, D-mannitol, and D-lactose), amino acids (e.g. L-asparagine, L-arginine, and L-serine) and carboxylic acids (e.g. D-malic acid) but also phosphor containing compounds like glucose-1-phosphate or esters like pyruvic acid methyl ester (Figs. 4 and S9). The *Microcystis* exudates had only a minor impact on substrate utilization. *Sphingomonas*, in contrast, was only able to show metabolic activity with *Microcystis* exudate supplementation. Both the WT and the Δ*mcyB* mutant exudate enabled substrate utilization on polymeric substrates (α-cyclodextrin, Tween-40, and Tween-80) and carbohydrates (D-cellobiose and N-acetyl-D-glucosamine). The growth promotion was much more pronounced with WT exudates, where higher mean well color values were reached compared to the Δ*mcyB* mutant exudates, supporting the hypothesis that the *Sphingomonas* prefers MC+ conditions (Figs. 4, S10). The *Flavobacterium* isolate, however, was not able to show metabolic activity in any condition, suggesting that other factors are needed for it to be metabolically active (Figs. 4, S11). We frequently observed that *Flavobacterium* could not grow in liquid culture; growth was only observed on agar plates. Here, biofilm formation or surface attachment might play an important role to stimulate growth and metabolism. Next, the three selected heterotrophs were studied through whole genome sequencing, using short-read BGI DNA nanoball sequencing (DNBSEQ) and subsequent annotation with bakta [45] and RASTtk [46] analysis. During the course of this analysis, the strains were named *Agrobacterium* sp. UP1, *Flavobacterium* sp. UP2, and *Sphingomonas* sp. UP3. In agreement with the EcoPlate™ analysis, the genome sequence of the *Agrobacterium* sp. UP1 revealed the broadest capabilities to utilize carbohydrate substrates including monosaccharides, carboxylic acids and sugar alcohols, among the three isolates. We could not identify pathways for nitrogen fixation in *Agrobacterium* sp. UP1. The genome of *Flavobacterium* sp. UP2 showed the fewest possibilities for utilizing carbohydrates. In particular, we compared the genomic potential to utilize 2-P-glycolate and glycolate, which are among the major dissolved organic carbon species in exudates of phototrophic microorganisms. KEGG pathway analysis revealed that both, *Sphingomonas* sp. UP3, and *Agrobacterium* sp. UP1 encode enzymes required for glycolate oxidation, as indicated in the KEGG pathway module “photorespiration” (Fig. 5, Table S7), whereas *Flavobacterium* sp. UP2 did not encode enzymes for the conversion of glycolate or glyoxylate (Table S7). This suggests that both *Agrobacterium* sp. UP1 and *Sphingomonas* sp. UP3 have the potential to utilize (2-P)-glycolate released by *Microcystis* and either use it as their own substrate (commensal interaction) or to convert it and return glycerate and CO_2_ back to *Microcystis* (mutualistic interaction), whereas *Flavobacterium* sp. UP2 was found to be uniquely equipped with a bicarbonate transporter (Fig. 5).

Moreover, we identified differences in the motility potential of the three heterotrophs. Both *Agrobacterium* sp. UP1 and *Sphingomonas* sp. UP3 encode the necessary components of the flagellar biosynthesis apparatus and one or two copies of the chemotaxis regulator CheY, respectively, indicating full capabilities for flagellar motility (Fig. 5, Tables S8 and Table S10). *Flavobacterium* sp. UP2, again, lacks this ability and only encodes three gliding motility-associated ABC transporter ATP-binding proteins and one putative archaeal flagellar protein (Fig. 5, Table S9). Taken together, the EcoPlate™ analysis, *Microcystis* exudate supplementation and genome sequencing indicate that both *Agrobacterium* sp. UP1 and *Sphingomonas* sp. UP3 are well equipped for cross-feeding with *Microcystis*. As *Sphingomonas* sp. UP3 was only able to grow with *Microcystis* exudate supplementation, it may more strongly rely on glycolate oxidation than *Agrobacterium* sp. UP1 and may require assistance from *Microcystis* for the degradation of complex polymers like Tween and α-cyclodextrin. In contrast, we were unable to gather any concrete evidence regarding the basis of the interaction between *Microcystis* and *Flavobacterium* sp. UP2 and therefore cannot explain why *Flavobacterium* sp. UP2 showed growth in the MC- community specifically.

We could not find hints for enzymatic MC-degradation via the *mlrA-D* gene cluster [47], as these genes were not identified among the genomes of the three heterotrophs. Because alternative routes for MC degradation have been shown to be environmentally relevant [48], we set out to evaluate whether the three selected heterotrophs employ other routes for MC degradation. We could not find any evidence that MC itself was metabolized or degraded, as MC accumulated in similar amounts both intracellularly and extracellularly in co-cultures of *M. aeruginosa* PCC 7806 and the three selected heterotrophic bacteria compared to a parallel grown monoculture (Fig. S12).

## Discussion

It has been increasingly recognized that *Microcystis* and its phycosphere bacteria must be regarded as holobiont, with numerous indications for a high specificity of the interactions of *Microcystis* and its microbiome and even evidence for a co-evolution of individual traits [7, 8, 14, 19, 49]. Yet, the possible role of MC has so far only been touched upon [20]. This is mainly due to that fact the ability to produce MC cannot be clearly assigned to specific *Microcystis* oligotypes [19, 50]. Additionally, most of the studies are still being carried out with bulk *Microcystis* samples, which do not allow discrimination between different genotypes [7, 13]. Although *Microcystis* single colonies have been used to study the *Microcystis* phycosphere composition [14, 19, 32], our independent single colony analysis allowed a more exclusive focus on MC without variation in seasonality, weather conditions, temperature, and nutrient concentrations. This should create a basis for the design of the subsequent synthetic community study and ultimately assist in gaining an integrative functional understanding of how MC contributes to the structure of the *Microcystis* phycosphere.

The *Microcystis* single colony analysis initially confirmed many known findings on the composition of the *Microcystis* phycosphere. In particular, we were able to observe many taxa that have already been described for bulk *Microcystis* samples or single colonies (e.g. *Flavobacterium*, *Roseomonas, Sphingomonas*, *Bradyrhizobium*, *Phenylobacterium*) [12, 20, 32, 37, 51], and we also did not observe a *Microcystis* core community [32]. Neither were we able to detect taxa exclusively occurring in MC+ or MC- colonies [19]. Yet, MC had a negative impact on community richness and certain taxa preferably occurred in MC+ or MC- colonies. According to the LEfSe analysis, the genera *Tabrizicola*, *Phenylobacterium,* and *Microscillaceae* were more prevalent in the MC+ colonies. *Tabrizicola* is a genus belonging to the *Rhodobacteraceae* family of aerobic anoxygenic phototrophs (AAP) that was recently associated with complementary nutrient recycling in blooms [52]. *Phenylobacterium* was previously described to promote the dominance of MC-producing over non-producing *Microcystis* [20]. The genera *Cutibacterium* and *Streptococcus*, in contrast, which were more strongly associated with MC- colonies, are rather atypical for *Microcystis* microbiomes. Their prevalence in MC- colonies may reflect the overall greater species richness in MC- colonies. However, this might also indicate that MC itself or the MC-dependent phenotypic differences of *Microcystis* act as a selective filter in the *Microcystis* phycosphere. However, because most of the MC+ preferring genera such as *Tabrizicola*, *Phenylobacterium,* and *Microscillacae* appear in co-occurrence networks of both colony types and only the individual ASVs differ, functional traits that are not part of the core genome of these genera are probably relevant for the MC preference. According to our study, genus assignment is therefore not a good indicator for MC+ or MC- colony specificity.

Individual *Microcystis* colonies differ in a large number of functional traits, such as their sheath properties and inorganic carbon adaptation, which poses a great challenge to capture the distinct influence of MC. The synthetic community experiment with the MC-producing strain *M. aeruginosa* PCC 7806 and its Δ*mcyB* mutant was therefore designed to reduce complexity. However, focusing the analysis on the factor MC using laboratory strains introduces a bias that can have a significant influence on the assembly of the microbial community. *M. aeruginosa* PCC 7806 grows in single-celled conditions and has reduced mucilage compared to field colonies. This major difference probably affects the ability to interact physically in particular. We therefore assume that the synthetic laboratory approach mainly promotes heterotrophic bacteria whose interaction with *Microcystis* is based on chemical interactions. Furthermore, the synthetic study is biased by the cultivability of isolates. We aimed to minimize this drawback by using CYA agar for isolation, favoring bacteria that rely on interactions with cyanobacteria or are able to utilize the degrading cyanobacterial biomass as their substrate, as described in Berg, *et al* [12]. The fact that the three selected isolates that grew exceptionally well in the consortium are either very often associated with *Microcystis* (*Sphingomona*s, *Flavobacterium*) or occasionally co-occurring with high abundance (*Agrobacterium*) indicates a specificity of the interactions that at least partially reflects the field situation. Since *Agrobacterium* is predominantly a terrestrial bacterium and is associated with *Microcystis* somewhat less frequently, it is possible that the fact that it has established itself as the dominant partner in the consortium is due to the single celled state of the laboratory strains. The strong mutual influence of the partners in our phototroph-heterotroph consortia is already reflected in the growth curves of the communities, which indicate a real tug-of-war between the partners (Figs. 2 and S7). In the ultimately stable consortium, the interactions appear to be mainly mutualistic, as the three heterotrophic isolates and also *Microcystis* itself grew during this period.

For the bacteria of the *Microcystis* phycosphere, a major role through complementary nutrient recycling is being discussed [7, 52]. We therefore examined the extent to which cross-feeding could contribute to a mutualistic relationship between the partners for the three selected organisms *Agrobacterium*, *Sphingomonas* and *Flavobacterium*. Based on metabolic profiling and KEGG pathway analysis, we observed a complementary metabolic potential, especially for *Agrobacterium* and *Sphingomonas,* in particular with regard to their ability to perform glycolate oxidation. Because many *Microcystis* strains have a weak carbon concentrating mechanism (CCM) [53] and inorganic carbon is a limited resource in blooms [54], we also assume that a major contribution of the selected heterotrophic bacteria is the supply of respiratory CO_2_ to *Microcystis* (Fig. 5).

The fact that *Sphingomonas* grew better with WT exudates suggests that MC plays an active role especially in cross-feeding between *Microcystis* and *Sphingomonas*. It has been known for some time that MC has a very prominent effect on the inorganic carbon metabolism of *Microcystis* and, in particular, influences the accumulation of RubisCO products [26, 27]. It is therefore possible that MC+ conditions could favor nutrient recycling and, in turn, the interaction with *Sphingomonas*. Our analysis showed that in the presence of MC, *Sphingomonas* was able to utilize more carbon sources, including complex substrates such as Tween-40, Tween-80, and α-cyclodextrin. This further suggests that *Microcystis* could potentially stimulate the degradation of these complex substrates. *Sphingomonas* is one of the bacterial genera frequently associating with *Microcystis* and is mainly studied in connection with the degradation of MC [47, 55]. However, the strain selected in this study is not equipped with the known MC degradation enzymes *mlrA-D*. We also could not find evidence for alternative routes of MC degradation (Fig. S12).

When looking at the co-occurrence networks of MC+ and MC- colonies, it is noticeable that one individual *Sphingomonas* ASV is linked with MC- colonies suggesting that also for this genus functional traits that are not part of the genus’ core genome are decisive for the interaction. Indeed, pathways such as MC degradation and glycolate oxidation are only sporadically encoded in *Sphingomonas* genomes and could influence the interaction in different ways. In agreement with this hypothesis, Berg et al. have already shown that different *Sphingomonas* isolates had diverse and even opposite outcomes on the growth of *Microcystis* using a cultivation-based approach [12].

A similar observation was also made for *Flavobacteria*, which are also frequently co-associated with *Microcystis* blooms. Here too, either growth-promoting or growth-inhibiting influences on *Microcystis* were detected [12], and *Flavobacteria*-ASVs also occur in both MC+ and MC- colonies in our co-occurrence networks (Fig. 1A). In our synthetic community study, the selected *Flavobacterium* isolate showed a clear preference for the Δ*mcyB* mutant strain. However, neither the metabolic profiling nor the genome sequence provides information on traits that could be pivotal for the interaction with *Microcystis* or explain the Δ*mcyB* preference. It is possible that the interaction of the partners in this case requires interaction pathways that cannot be reproduced by the addition of exudates. As *M. aeruginosa* PCC 7806 and its Δ*mcyB* mutant are known to differ in their cell surface characteristics, e.g. in the expression of the cell surface glycoprotein MrpC, a preferential interaction with one of the genotypes could also be rooted in alterations of cell–cell interaction characteristics.

In summary, both the field experiment and the synthetic community experiment suggest that MC has an influence on the structure of the phycosphere microbiome of *Microcystis*, with a more pronounced effect in the synthetic community analysis. We were able to show that MC probably influences cross-feeding between the strains through its influence on inorganic carbon metabolism and/or by promoting the degradation of complex substrates. The study thus further emphasizes previous findings on the role of *Microcystis* phycosphere bacteria in complementary nutrient recycling and highlights the great importance of strain-specific traits for the nature of interactions and the response to MC production.

### Data deposition

The raw sequence reads obtained in this study have been deposited in the Sequencing Read Archive under BioProject number PRJNA1148368. Remaining data and scripts can be obtained at https://gitup.uni-potsdam.de/ag_mibi/NSMCA.

## Supplementary Material

Supplementary_information_1223_ISMEComm_ycae170

Supplementary_tables_ycae170
