# Supplementary material for: Microcystin shapes the Microcystis phycosphere through community filtering and by influencing cross-feeding interactions
Source: ISME Commun. 2024 Dec 24;5(1):ycae170. doi: 10.1093/ismeco/ycae170 (PMC11748430; doi:10.1093/ismeco/ycae170)
Supplement: Supplementary_information_1223_ISMEComm_ycae170 [file supplementary_information_1223_ismecomm_ycae170.pdf]

Supplementary information to:

Microcystin shapes the *Microcystis* phycosphere through community filtering and by influencing cross-feeding interactions

Rebecca Große[1], Markus Heuser[1], Jonna E. Teikari[2,3], Dinesh K. Ramakrishnan[4], Ahmed Abdelfattah[4], Elke Dittmann[1]

Table of contents:

Supplementary methods and materials

Method S1: Procedure for isolation of single colonies *3*
Method S2: Heterotrophic bacterial strain identification: PCR and amplicon purification. *3*
Method S3: Pre-culture cultivation conditions for Co-cultivation experiment *3*
Method S4: Small scale co-cultivation experiment *4*
Method S5: DNA isolation from synthetic communities *6*
Method S6: Processing of the single colonies data set *6*
Method S7: 16S-rRNA amplicon sequencing and microbial community analysis *7*
Method S8: EcoPlates experimental procedure *8*
Method S9: Genome sequencing and annotation of selected heterotrophs *8*
References *9*

Supplementary tables

Table S1: ASV table of 29 single Microcystis colonies *10*
Table S2: Relative presence of ten most prevalent taxa in 29 single Microcystis colonies *11*
Table S3: Overview of heterotrophic bacterial strains and media *12*
Table S4: Mean relative abundances of Microcystis in the synthetic communities *13*
Table S5: Settings for the ESI source used in this study. *13*
Table S6: Settings for DDA, filters and MS2 used in this study. *14*

Supplementary figures

Figure S1: Rarefaction curves *15*
Figure S2: Gallery of representative Microcystis colonies *16*
Figure S3: Agarose gel electrophoresis of mcyA-Cd fragment amplicons *17*
Figure S4: Co-occurrence networks of mcyA(+) and mcyA(-) bacterial communities *18*
Figure S5: Differential abundance analysis of taxa on genus level of single Microcystis colonies *19*
Figure S6: Physiological state of the cultures from the co-cultivation experiment after four weeks of cultivation (T4) *20*
Figure S7: Relative abundances of heterotrophic bacterial genera in SynCom experiment .. *21*
Figure S8: LEfSe analysis of SynCom experiment *22*
Figure S9: EcoPlateTM substrate utilization test of Agrobacterium sp. UP1. .... *23*

Figure S10: EcoPlateTM substrate utilization test Sphingomonas sp. UP3 24
Figure S11: EcoPlateTM substrate utilization test of Flavobacterium sp. UP2 25
Figure S12: Comparative chromatograms of intracellular and extracellular extracts of M. aeruginosa PCC 7806 and ΔmcyB mutant monocultures (WT and MUT axenic) and cocultures 26

Supplementary methods and materials

Method S1: Procedure for isolation of single colonies

Microcystis colonies were sampled from the water surface, using a plankton net (mesh size: 70 µm) and transferred into 50 mL Falcon tubes. During transportation the samples were kept in the dark at 4°C. Colony isolation was done under the stereo microscope Stemi 305 (Zeiss, Oberkochen, Germany) following the "Isolation Using Micropipettes" method described by Kurmayer, R. et al. [1] within 36 h after sampling and were kept at continuous light (50 µmol photons $m^{-2}s^{-1}$) during that period. Images of single colonies were acquired with the Zeiss Axiocam microscope camera. Single colonies were stored in 10 µL sterile H_2O at -20°C until DNA extraction.

Method S2: Heterotrophic bacterial strain identification: PCR and amplicon purification

A single colony was picked from an agar plate and resuspended in 20 µL of sterile MilliQ water. 10 µL were used immediately to inoculate a fresh agar plate to ensure that the taxon identified after sequencing and the subsequent bacterial cultures are of the same origin. 16S-rRNA gene amplicons were generated using the 16S_27_FW (5'-AGAGTTTGATCCTGGCTCAG-3') and 16S_1492_RV (5'-GGTTACCTTGTTACGACTT-3') primers [2] in a PCR with Phusion™ High Fidelity DNA polymerase (Thermo Fisher Scientific, Waltham, MA, USA) and 1.5 µL of the bacterial suspension as template. PCR cycler settings were set according to the manufacturers protocol with the following changes: Initial denaturation step was extended to 10 min to break down the bacterial cells. PCR products were purified with the GeneJET PCR purification kit (Thermo Fisher Scientific) and sequenced using Sanger sequencing approach done by LGC genomics (LGC Ltd, Teddington, England), using the same primer pair. After Sanger sequencing, 16S-rRNA amplicon sequences with a minimal length of 415 bp and a quality score ≥ 40 were used in a BLAST nucleotide search. The extract_regions_16s tool (https://github.com/AlessioMilanese/extract_regions_16s) was used to obtain the V6 regions. V6 regions were copied to a FASTA file and used to construct a Maximum-likelihood phylogenetic tree with bootstrapping of 100 iterations using the Tamura 3-parameter model in the MEGA X software (version 11.0.13).

Method S3: Maintenance cultivation conditions for Co-cultivation experiment

Maintenance cultures of cyanobacterial strains *Microcystis aeruginosa* PCC 7806 wild type (WT) and MC-LR deficient Δ*mcyB* mutant were grown in BG11 medium at RT [3]. For the

Δ*mcyB* mutant 5 µg/mL chloramphenicol were added to the BG11 medium. Culture flasks were shaken once a day. To assure exponential growth, cultures were passaged when the OD_{750} of cultures reached 1.0 and OD_{750} of fresh cultures was adjusted to approximately 0.2. Axenicity of cultures was monitored regularly by streaking a small sample on R2A agar plates and via microscopy.
Heterotrophic bacterial precultures were grown on R2A agar plates, except strains ENV4 and ENV3 were grown on Marine Agar (detailed information in Table S3), in the dark at approximately 25°C. Passaging of heterotrophic cultures was done monthly. For the experiments, only bacteria were used that were passaged not more than 5 times.

Method S4: Small scale co-cultivation experiment

M. aeruginosa PCC 7806 WT and Δ*mcyB* mutant were cultivated in 50 mL Erlenmeyer culture flasks together with a small community consisting of *Sphingomonas*, *Flavobacterium* and *Agrobacterium* at identical conditions described in the co-cultivation experiment. Bacterial cell suspensions were diluted so that the final amount of each heterotroph isolate should have a cell number of 1/3rd of the Microcystis cell number (~$2*10^6$). 20 mL samples were taken after 14 days of cultivation. Cells were pelleted by centrifugation (4700*g, 10 min, RT) resuspended in distilled water and lysed using ultrasonication (10 min, 2 sec on-off-interval, 60% amplitude). Culture supernatants and cell lysate were extracted using SPE C-18 cartridges (Chromafix C18ec, 731805, Macherey Nagel) and filtered (Chromafil Xtra H-PTFE-20/13, 729256, Macherey Nagel) prior to injection into the HPLC or LC-MS system, respectively.

HPLC analysis was performed on a Thermo Fisher Vanquish Core (VC-P20-A) equipped with a Split Sampler (VC-A12-A, Thermo Fisher), a diode array detector (VF-D11-A, Thermo Fisher), a column oven (VC-C10-A, Thermo Fisher) and a fraction collector (VF-F10-A, Thermo Fisher). For separation, a RP-C18 column (Hypersil GOLD, 250x4.6 mm, 5µ particle size, 25005-254630, Thermo Fisher) with a guard column (Hypersil GOLD, 10x4 mm, 5 µm particle size, 25005-014001, Thermo Fisher) and an Acetonitrile/Water gradient (Acetonitril Hypergrade LC-MS, 1000292500, VWR; LC-MS grade water, 83645.320, VWR) was used. Both mobile phase constituents contained 0.05% (v/v) TFA (Trifluoroacetic Acid LC-MS grade, 84868.180, VWR). The column was heated to 40°C in Still Air mode and the flow rate was 1 mL/min. The time program consisted of a 5 min isocratic (5% ACN) preconditioning step, followed by injection and the immediate start of a 5% to 95% ACN gradient over 20 min. Subsequently, another 4 min isocratic step (95% ACN) was used to elute remaining compounds

off the column. Then, the column was brought back to starting conditions by a short 95% to 5% CAN gradient over 1 min and another 2 min isocratic (5% ACN) step. Detection was done by simultaneous measurement of photometric absorption between 190 – 345 nm with a 4 nm bandwidth and 10 Hz sampling rate. Chromeleon 7.2.10 ES served as the CDS to inspect and analyze the data.

The same set of samples was further analyzed by UHPLC-MS/MS using the following instrumentation: Vanquish Flex (VF-P10-A, Thermo Fisher) equipped with a Split Sampler (VF-A10-A, Thermo Fisher), a diode array detector (VF-D11-A) and a column oven (VH-C10-A) connected to an ion trap high resolution mass spectrometer (Orbitrap Exploris 240, Thermo Fisher). For separation on the UHPLC system, a RP-C18 column (Kinetex C18, 50x2.1 mm, 1.7 µm particle size, 00B-4475-AN, Phenomenex) with a guard column (SecurityGuard ULTRA C18, 2.1 mm, AJ0-8782, Phenomenex) and an Acetonitril/Water gradient (same as above) was used. Both mobile phase constituents contained 0.1% FA (Formic Acid LC-MS grade, 84865.180, VWR). The column was heated to 40°C in Still Air mode and the flow rate was 0.5 mL/min. The time program consisted of a 2 min isocratic (5% ACN) step, followed by injection and another 1 min isocratic (5% ACN) step. Then, a 5% - 100% ACN gradient over 10 followed and remaining compounds were eluted off the column for 2 min at 100% isocratic flow. Afterwards, the column was brought back to starting conditions using a short 100% - 5% ACN gradient over 1 min. PDA detection was done by simultaneous measurement of photometric absorption between 190 – 800 nm with a 4 nm bandwidth and 10 Hz sampling rate.

To protect the mass spectrometer from high salt freight and strongly hydrophobic compounds, the LC flow was diverted into the waste for 0.5 min after injection and after 11.5 min during each run.

Samples were ionized in an Optamax NG ESI source (Thermo Fisher), using the settings shown in Table S5.

Data-dependent MS/MS acquisition was done in Cycle Time mode with 400 ms between each MS^1 survey scan. An in-detail listing of used settings is given in Table S6.

Advanced Peak Determination was turned on while Mild Trapping was turned off. No MS scans were recorded during time frames in which the LC flow was diverted into the waste.

The resulting raw data were converted into mzML file format using MSConvertGUI (v. 3.0.23194-4f3fc79). The “peakPicking” filter was applied with the parameter setting “vendor

msLevel=1-2”. Additionally, options were used as follows: Binary Encoding Precision of 64 bit, Write Index: yes, TPP compatibility: Yes. All other options were turned off.
Extracted ion chromatograms were built using a custom Python (v. 3.11.2) script that ran MassQL (v. 0.0.15) [4] queries against each individual mzML file, gathered and concatenated the results and stored them in CSV format for visualization in R (v. 4.4.1) using ggplot2 (v. 3.5.1) [5], ggpubr (v. 0.6.0) [6], tidyverse (v. 2.0.0) [7] and svglite (v. 2.1.3) [8]. The following query was used:

```
QUERY scansum(MS1DATA) FILTER MS1MZ=precursorMZ:TOLERANCEPPM=5
```

The term *precursorMZ* was programmatically replaced by either `981.5404` for DAsp3-MC-LR or `995.5560` for MC-LR, respectively.

Method S5: DNA isolation from synthetic communities

First, cells from 10 mL sample volume were harvested (4700*g, 10 min, 4°C), resuspended in 900 µL ATL buffer and added with 0.5 g sterile glass beads (0,5 mm) and 0.5 g sterile glass beads (0,1 mm), followed by a beating step at maximum speed on a vortex for 45 seconds. Cells were then immediately incubated at 56°C for 30 min and beat again for 45 sec at maximum speed. Then, 100 µL Proteinase K were added and cells were incubated at 56° (2h). Beads were collected at the bottom of the tube via centrifugation (2 min, 1000*g, 4°C), bead-free supernatant was transferred to a fresh tube and centrifuged again (1 min, max speed, RT). Subsequently, 650 µL of the supernatant were mixed with 650 µL of non-denatured ethanol and transferred to a mini spin column. DNA purification was done according to the manufacturers protocol with a final elution step in 150 µL DNA-free water. All steps were performed under sterile conditions.

Method S6: Processing of the single colonies data set

The chemotype of 49 single Microcystis colonies were tested for the presence/absence of an NRPS gene from the MC biosynthetic gene cluster *mcyA* using the primer pair *mcyA*-Cd-1R (5′-AAAAGTGTTTTATTAGCGGCTCAT-3′) and *mcyA*-Cd-1F (5′-AAAATTAAAAGCCGTATCAAA-3′) [9]. PCR with Phusion™ High Fidelity DNA polymerase (Thermo Fisher Scientific) was carried out using 1 µL of the extracted DNA as template. PCR reaction was run according to the manufacturers protocol. Amplicons of the

expected band size (291 bp) were observed. 13 colonies that exhibit a strong PCR band signal were classified as *mcyA*(+). 18 colonies that showed no PCR band signal were classified as *mcyA*(-) colonies (Figure S3). Twenty-one colonies with weak PCR signal were considered "mixed" type (not classified (NC)) and excluded from further analysis. Additionally, one *mcyA*(+) and one *mcyA*(-) colony sample (Sample 19 and Sample 20, respectively) were excluded from the analysis due to atypical high abundances of Vampirivibrionia Class, that was not observed in any other sample. The remaining dataset of 12 *mcyA*(+) and 17 *mcyA*(-) colonies were used for the subsequent analysis.

Method S7: 16S-rRNA amplicon sequencing and microbial community analysis

Microbiome analysis was performed based on the sequencing of the 16S-rRNA V3-V4 gene region, using the universal primer pair 806R (5'-GGACTACHVGGGTWTCTAAT-3') and 341F (5'-ACTCCTACGGGAGGCAGCAG-3'). Single colony sequencing was done on the Illumina MiSeq PE300 platform (BGI Tech Solutions Co., Hong Kong, China). For the synthetic community experiment the DNBSEQ platform was used (BGI Tech Solutions). Raw reads were trimmed using cutadapt version 2.10 (parameter settings: --quality-cutoff 21 \ --minimum-length 213) [10] and quality checked using FASTQC and MULTIQC [11; 12]. High quality reads were merged using the DADA2 pipeline [13] with taxonomic classification based on SILVA database version 138.1 [14]. Statistical analysis of sequence data was done in RStudio (2024.04.2+764 Chocolate Cosmos) with R version 4.3.2, using the microViz package (https://david-barnett.github.io/microViz/) (49). Relative abundances were calculated using the "compositional" approach. Richness was estimated using the exp_shannon function. Linear discriminant analysis effect size (LEfSe) analysis was done with the microbiomeMarker package with the following parameters: $p < 0.05$ (Kruskal-Wallis-Test), logLDA cutoff = 3, CSS normalization [15]. Microbial association networks were constructed using CoNet with its default parameters and visualized in Cytoscape version 3.10.2 [16]. Taxa with co-presence were filtered using the co-presence parameter with a minimum number of 3 connections including between *Microcystis* and heterotrophic bacteria and between heterotrophic bacteria with heterotrophic bacteria.

Method S8: EcoPlates experimental procedure

Bacterial strains (*Agrobacterium, Flavobacterium, Sphingomonas*) were grown on R2A agar plates at 25°C until sufficient biomass was yielded. In order to test the influence of cyanobacterial exudates on bacterial growth and substrate utilization, 200 mL culture supernatant from parallelly grown maintenance cultures of *M. aeruginosa* PCC 7806 (WT) and Δ*mcyB* mutant was obtained. The cultures were centrifuged (4700 rpm, 10 min, RT) and subsequently the exudates containing supernatant was collected, sterile filtered (0.2 µm) and stored at 4°C until further use. For the substrate screening experiment, bacterial biomass was scraped off the agar plates and resuspended either in sterile filtered WT/ Δ*mcyB* mutant exudates or 0.9%-NaCl solution (according to the manufacturers protocol). Turbidity of the suspension was adjusted to 60%. For each bacterial strain, one plate was prepared for each condition, resulting in a total of 9 plates. Plates were incubated at 25°C for 192 h in the OmniLog 50 incubator using the OmniLog Data Collection Software 3.0. Raw data were transformed to CSV-format with the Data Analysis Software 1.7. Heatmap and kinetic plots were created in RStudio (2024.04.2+764 Chocolate Cosmos) with R version 4.3.2 using the ggplot2 package [6].

Method S9: Genome sequencing and annotation of selected heterotrophs

For whole genome sequencing of the three selected heterotrophic bacterial strains (*Agrobacterium sp* UP1, *Flavobacterium sp.* UP2 and *Sphingomonas sp*. UP3), bacteria were freshly taken from cryo-preserved culture and grown on R2A agar plates until sufficient biomass was yielded. The cells were harvested and diluted in ATL buffer (Qiagen). DNA was extracted according to the protocol for the synthetic communities mentioned above. High quality genomic DNA was sequenced, using DNBSEQ method (BGI Tech Solutions Co.). Raw reads were quality checked using FASTQC [11]. Genomes were assembled using shovill (https://github.com/tseemann/shovill) and assemblies were inspected and evaluated using Bandage [17] and Quast [18]. Assembled genomes were annotated using the bakta pipeline [19] and the annotation server tool RAST following the RASTtk annotation scheme with default settings [20]. Subsequent KEGG comparison and genome browsing was done with the SEED Browser [21].

References

1 Kurmayer R, Sivonen K, Wilmotte A *et al. Molecular Tools for the Detection and Quantification of Toxigenic Cyanobacteria*. Wiley, 2017.

2 Atilano ML, Glittenberg M, Bahuguna S *et al. Drosophila Toll links systemic immunity to long-term intestinal function*. bioRxiv.com.

3 Stanier RY, Deruelles J, Rippka R *et al.* Generic Assignments, Strain Histories and Properties of Pure Cultures of Cyanobacteria. *Microbiology* 1979;**111**:1–61.

4 Jarmusch AK, Aron AT, Petras D *et al. A Universal Language for Finding Mass Spectrometry Data Patterns*, 2022.

5 Wickham H. *ggplot2: Elegant Graphics for Data Analysis*. Springer-Verlag NewYork, 2016.

6 Alboukadel Kassambara. *ggpubr: 'ggplot2' Based Publication Ready Plots*. https://rpkgs.datanovia.com/ggpubr/.

7 Wickham H, Averick M, Bryan J *et al.* Welcome to the Tidyverse. *JOSS* 2019;**4**:1686.

8 Wickham H, Henry L, Pedersen T, Luciani T, Decorde M, Lise V. *svglite: An 'SVG' Graphics Device_*, 2023.

9 Hisbergues M, Christiansen G, Rouhiainen L *et al.* PCR-based identification of microcystin-producing genotypes of different cyanobacterial genera. *Arch Microbiol* 2003;**180**:402–10.

10 Martin M. Cutadapt removes adapter sequences from high-throughput sequencing reads. *EMBnet j* 2011;**17**:10.

11 Andrews S, Krueger F. *FastQC: a quality control tool for high throughput sequence data.* Babraham, UK, 2012.

12 Ewels P, Magnusson M, Lundin S *et al.* MultiQC: summarize analysis results for multiple tools and samples in a single report. *Bioinformatics* 2016;**32**:3047–48.

13 Callahan BJ, McMurdie PJ, Rosen MJ *et al.* DADA2: High-resolution sample inference from Illumina amplicon data. *Nat Methods* 2016;**13**:581–83.

14 Quast C, Pruesse E, Yilmaz P *et al.* The SILVA ribosomal RNA gene database project: improved data processing and web-based tools. *Nucleic Acids Res* 2013;**41**:D590-6.

15 Cao Y, Dong Q, Wang D *et al.* microbiomeMarker: an R/Bioconductor package for microbiome marker identification and visualization. *Bioinformatics* 2022;**38**:4027–29.

16 Faust K, Raes J. CoNet app: inference of biological association networks using Cytoscape. *F1000Res* 2016;**5**:1519.

17 Wick RR, Schultz MB, Zobel J *et al.* Bandage: interactive visualization of de novo genome assemblies. *Bioinformatics* 2015;**31**:3350–52.

18 Gurevich A, Saveliev V, Vyahhi N *et al.* QUAST: quality assessment tool for genome assemblies. *Bioinformatics* 2013;**29**:1072–75.

19 Schwengers O, Jelonek L, Dieckmann MA *et al.* Bakta: rapid and standardized annotation of bacterial genomes via alignment-free sequence identification. *Microb Genom* 2021;**7**.

20 Aziz RK, Bartels D, Best AA *et al.* The RAST Server: rapid annotations using subsystems technology. *BMC Genomics* 2008;**9**:75.

21 Overbeek R, Begley T, Butler RM *et al.* The subsystems approach to genome annotation and its use in the project to annotate 1000 genomes. *Nucleic Acids Res* 2005;**33**:5691–702.

Supplementary Tables

Table S1: ASV table of 29 single *Microcystis* colonies. ASVs shown were assigned to the genus *Microcystis*-PCC7941 with more than 1000 reads in at least one sample. Green = colonies with dominant ASV2, blue = colonies with dominant ASV3, red = colonies with no dominant ASV, yellow = colonies with dominant ASV1.

Sample No	Toxicity	ASV1	ASV2	ASV3	ASV4
10	T	0	12613	0	0
13	T	0	11037	0	0
15	T	0	7058	0	0
17	T	0	8894	0	0
23	NT	0	1251	0	0
25	NT	0	9429	0	0
26	T	0	10924	0	0
27	NT	0	14687	0	0
30	NT	0	10026	0	0
31	T	0	9396	0	0
36	NT	0	10842	0	0
44	NT	0	9942	0	0
21	NT	745	0	12939	0
7	T	2681	6694	0	0
47	NT	3676	0	11808	0
38	NT	5423	2735	0	0
45	NT	7766	2688	0	361
8	T	8132	9007	0	0
43	NT	9638	1181	0	1227
24	NT	8254	0	0	0
11	T	9257	0	0	0
48	NT	9498	814	0	0
32	NT	10254	0	0	0
6	NT	12973	0	0	0
28	NT	13163	0	0	0
1	T	15232	0	0	0
2	T	15572	0	0	503
39	NT	6186	0	0	0
16	T	7616	0	0	389

Table S2: Relative presence of ten most prevalent taxa in 29 single *Microcystis* colonies. Taxonomic resolution is the genus level.

Genus	Presence in percent of colonies
Roseomonas	75,9%
Microscillaceae Family	75,9%
Vibrio	72,4%
Cutibacterium	69,0%
Pelomonas	69,0%
Tabrizicola	69,0%
Phenylobacterium	62,1%
Flavobacterium	62,1%
Staphylococcus	55,2%
UKL13-1	48,3%

Table S3: Overview of heterotrophic bacterial strains and media used for the assembly of the heterotrophic consortium to conduct the co-cultivation experiments.

Het ID	Genus	Source	Culture media
Het 1	*Exiguobacterium*	Environment: Lake Wublitz	R2A (DSMZ No 830)
Het 2	*Flavobacterium*	Environment: Lake Wublitz	R2A (DSMZ No 830)
Het 3	*Vogesella*	Environment: Lake Wublitz	R2A (DSMZ No 830)
Het 4	*Pseudomonas*	Environment: Lake Wublitz	R2A (DSMZ No 830)
Het 5	*Pseudomonas*	Environment: Lake Wublitz	R2A (DSMZ No 830)
Het 6	*Acinetobacter*	Environment: Lake Wublitz	R2A (DSMZ No 830)
Het 7	*Acinetobacter*	Environment: Lake Wublitz	R2A (DSMZ No 830)
Het 8	*Ideonella*	Environment: Lake Wublitz	R2A (DSMZ No 830)
Het 9	*Chryseobacterium*	Environment: Lake Wublitz	R2A (DSMZ No 830)
Het 10	*Pseudomonas*	Environment: Lake Wublitz	R2A (DSMZ No 830)
Het 11	*Chryseobacterium*	Environment: Lake Wublitz	R2A (DSMZ No 830)
Het 12	*Chryseobacterium*	Environment: Lake Wublitz	R2A (DSMZ No 830)
Het 13	*Acinetobacter*	Environment: Lake Wublitz	R2A (DSMZ No 830)
Het 14	*Roseomonas sp.*	Provided by AG Zedler, Friedrich-Schiller Universität Jena	R2A (DSMZ No 830)
Het 15	*Methylobacterium*	Provided by AG Zedler, Friedrich-Schiller Universität Jena	R2A (DSMZ No 830)
Het 16	*Thalassococcus*	Provided by AG Zedler, Friedrich-Schiller Universität Jena	Marine Medium (DSMZ No 123)
Het 17	*Ruegeria*	Provided by AG Zedler, Friedrich-Schiller Universität Jena	Marine Medium (DSMZ No 123)
Het 18	*Paracoccus sp*	Provided by AG Zedler, Friedrich-Schiller Universität Jena	R2A (DSMZ No 830)
Het 19	*Sphingomonas*	Lab strain: non-axenic *Synechocystis* PCC 6803 (RMA mutant)	R2A (DSMZ No 830)
Het 20	*Agrobacterium*	Lab strain: non-axenic *Synechocystis* PCC 6803 (RMA mutant)	R2A (DSMZ No 830)
Het 21	*Dietzia*	Lab strain: non-axenic *Nostoc punctiforme* PCC 73102	R2A (DSMZ No 830)

Table S4: Mean relative abundances of *Microcystis* in the synthetic communities at the respective timepoint (*n* = 3).

	***M. aeruginos*a WT**		***ΔmcyB* MUT**	
	**Mean relative abundance**	**Standard deviation**	**Mean relative abundance**	**Standard deviation**
**T0**	19,5%	NA	38,1%	NA
**T1**	82,0%	3,4%	82,1%	3,3%
**T2**	30,7%	8,3%	38,7%	6,3%
**T3**	62,5%	6,5%	52,3%	3,1%
**T4**	52,0%	13,6%	47,8%	11,6%

Table S5: Settings for the ESI source used in this study.

Setting	Value
**Ion Source Type**	H-ESI
**Spray Voltage (+)**	3500 V
**Sheath Gas**	40
**Aux Gas**	5
**Sweep Gas**	1
**Ion Transfer Tube Temperature**	300 °C
**Vaporizer Temperature**	350 °C

Table S6: Settings for DDA, filters and MS^2 used in this study.

Setting	Value
**MS^1 Full Scan**	
**Full Scan Properties**	
- **Resolution**	30000
- **Scan Range**	100 – 1500 m/z
- **RF Lens**	70 %
- **AGC Target**	Custom
o **Normalized AGC Target**	20
- **Maximum Injection Time Mode**	Custom
o **Maximum Injection Time**	54 ms
- **Microscans**	1
- **Data Type**	Centroid
- **Polarity**	Positive
- **Source Fragmentation**	No
**Filters for ion selection**	
**Intensity Threshold**	1E4
**Dynamic Exclusion**	
- **Exclude after n times**	1
- **Exclusion duration**	1.5 s
- **Mass Tolerance (Low)**	5 ppm
- **Mass Tolerance (High)**	5 ppm
- **Exclude Isotopes**	Yes
- **Single Charge state per precursor only?**	No
**Apex Detection Window**	30 %
**MS2 Scan**	
**Data-Dependent MS2 Scan Properties**	
- **Multiplex Ions**	No
- **Isolation Window**	1 m/z
- **Isolation Offset**	No
- **Collision Energy Type**	Normalized
- **HCD Collision Energy**	80 %
- **Resolution**	15000
- **Scan Range Mode**	Auto
- **AGC Target**	Custom
o **Normalized AGC Target**	20
- **Maximum Injection Time Mode**	Custom
o **Maximum Injection Time**	54 ms
- **Microscans**	1
- **Data Type**	Centroid

Supplementary figures

(A)

(B)

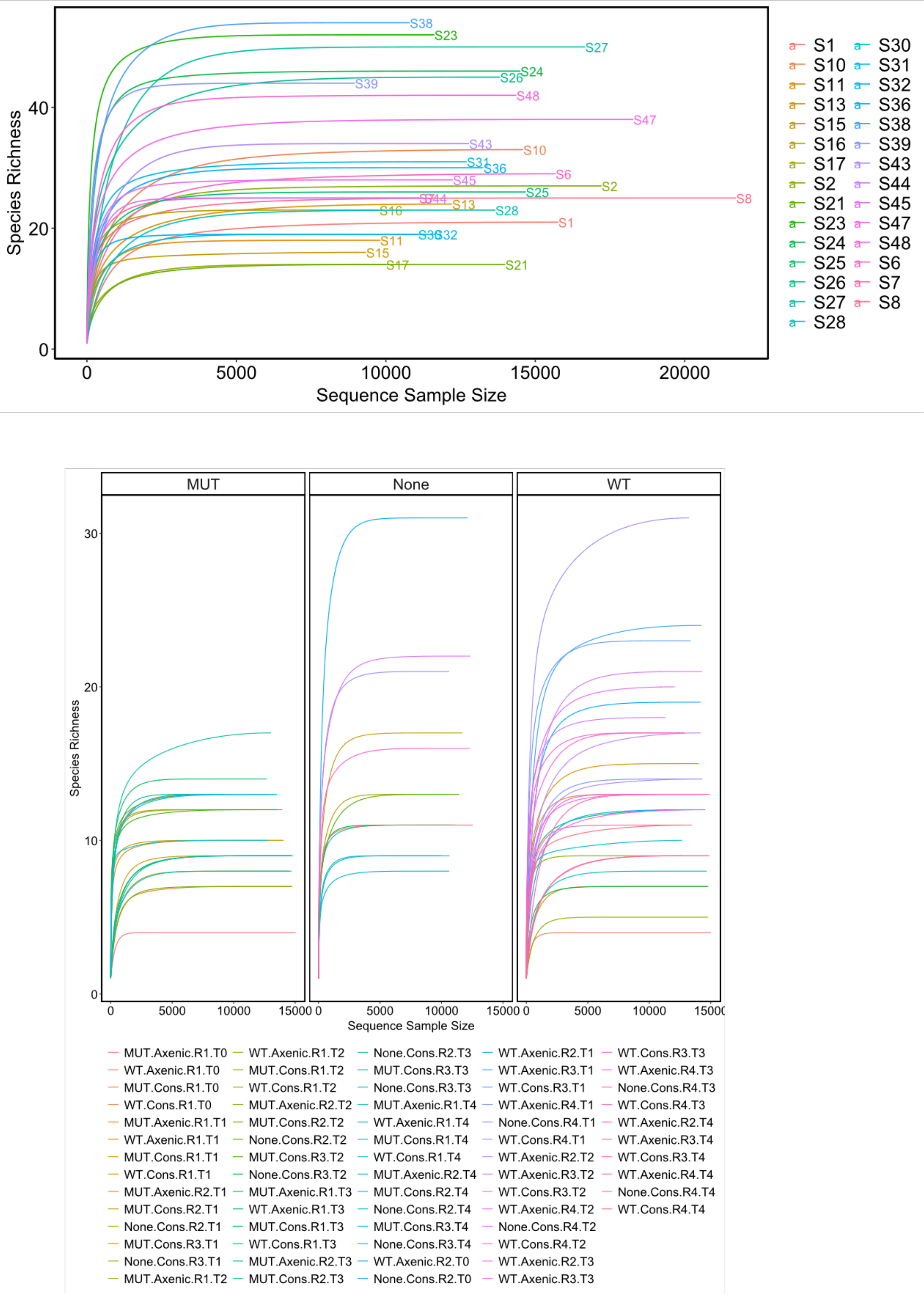

Figure S1: Rarefaction curves of (A) single colony sequencing and (B) co-cultivation experiment.

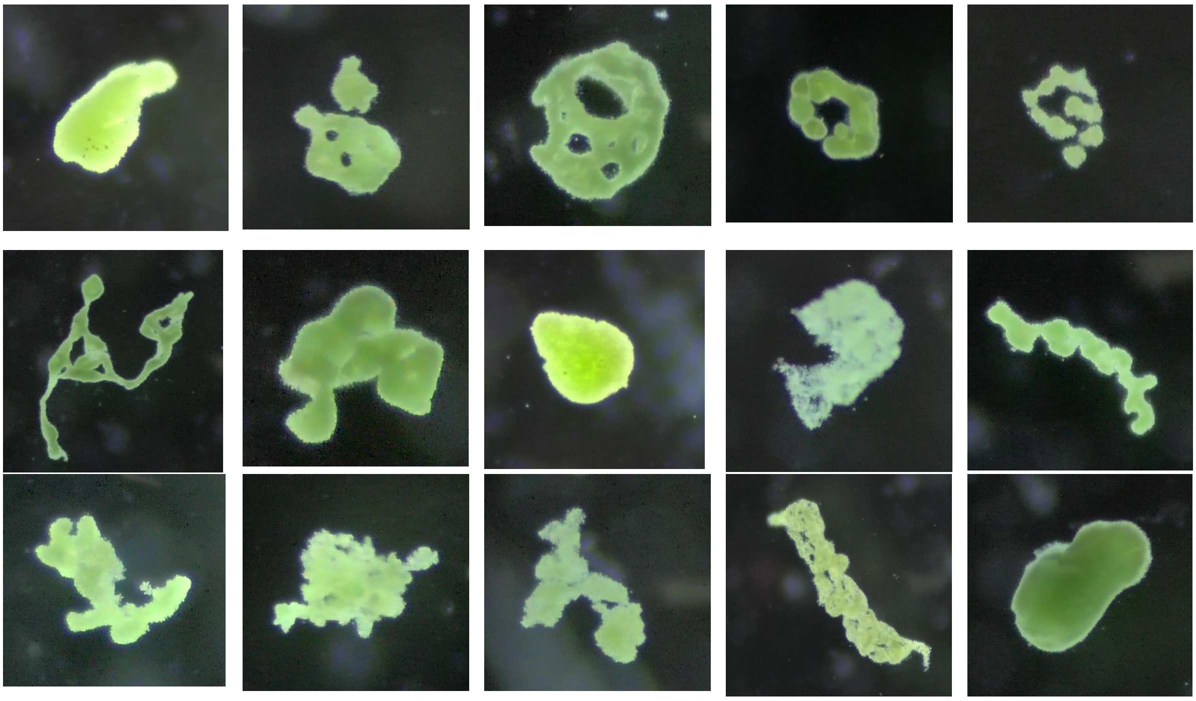

Figure S2: Gallery of representative *Microcystis* colonies collected from the Havel river in the Potsdam area (Tiefer See, 52.402987997097476, 13.079468904213506), multiple sampling time points during July and August 2023. Images acquired with Zeiss Stemi 305, equipped with external microscope camera, 10x magnification.

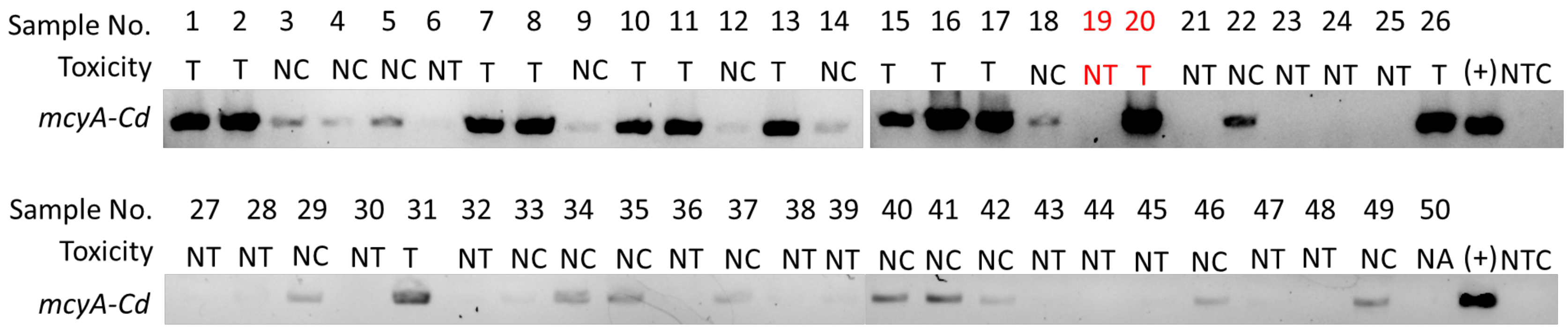

Legend

T	–	MC-producing (toxic)
NT	–	non-producing (non-toxic)
NC	–	excluded from analysis (not classified)
Red	–	excluded from analysis (unregular abundance of Vampirivibrionia class)
(+)	–	positive control (*M. aeruginosa* PCC 7806 gDNA)
NTC	–	negative control (no template control)

Figure S3: Agarose gel electrophoresis of *mcyA-Cd* fragment amplicons. DNA isolated from single *Microcystis* colonies was used as template. Band size of about 300 bp was confirmed with the GeneRuler Low Range DNA Ladder (Thermo Scientific). Colony enumeration and classification into toxic and non-toxic colonies is indicated by letters above each band.

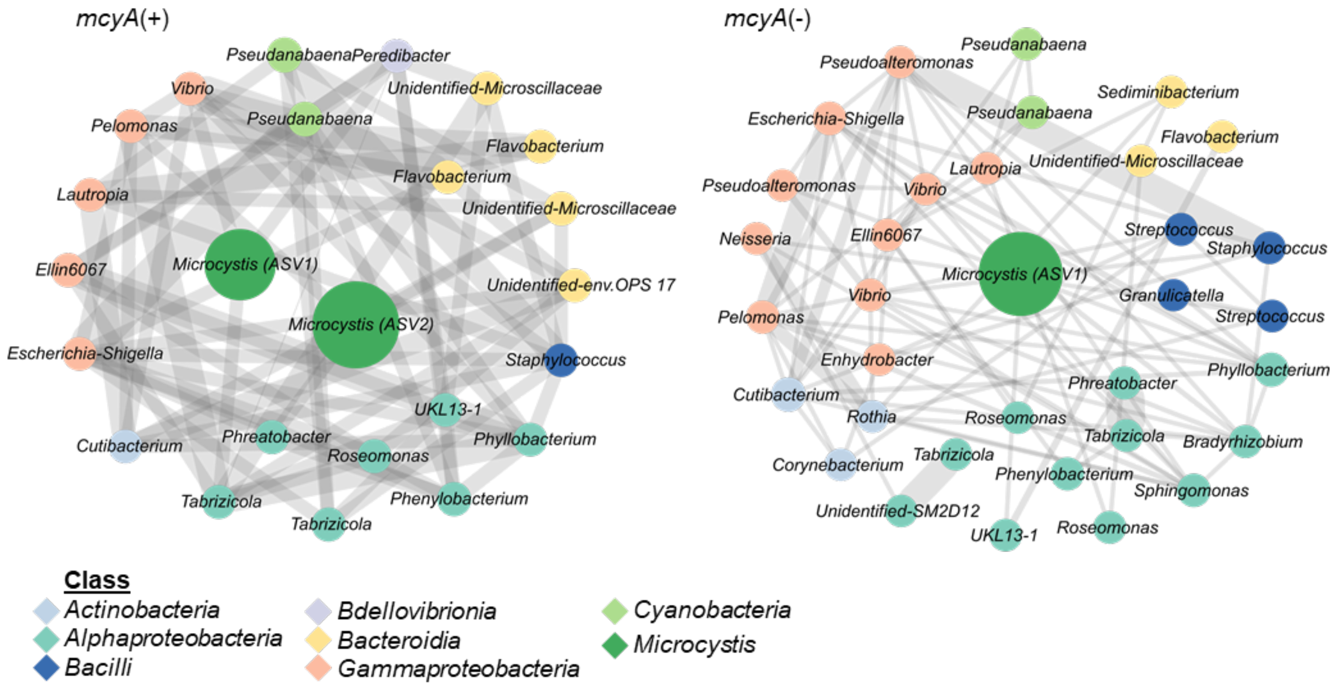

Figure S4: Co-occurrence networks of *mcyA*(+) and *mcyA*(-) bacterial communities on ASV level. Nodes were colored according to the respective taxonomic class, except *Microcystis* was colored according to genus. Node size reflects relative abundance. Edges show weighted positive co-presence correlation.

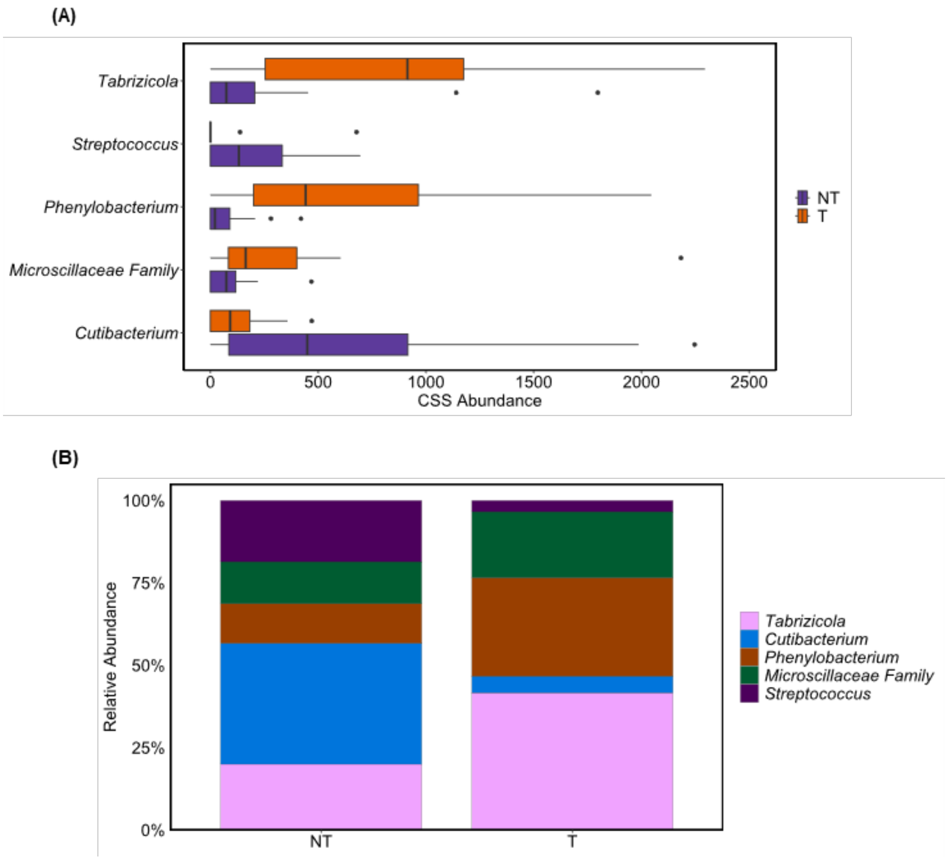

Figure S5: Differential abundance analysis of taxa on genus level of single *Microcystis* colonies. (A) Boxplot of CSS abundance of taxa scored by LEfSe analysis of *Microcystis* single colonies. (B) Mean relative abundances of taxa scored by LEfSe analysis. NT = non-MC-producing (MC-) (n = 17), T = MC-producing (MC+) (n = 12).

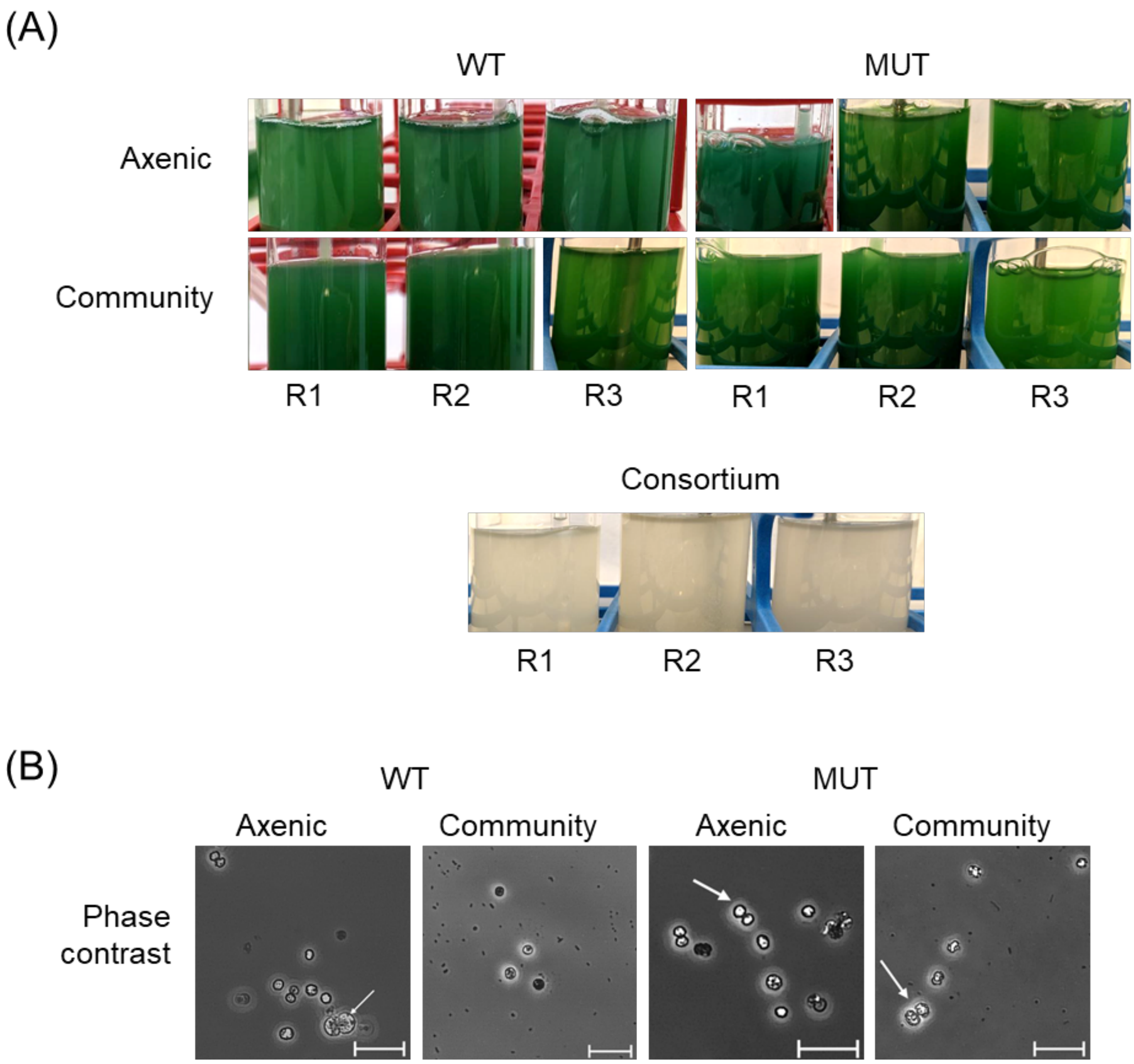

Figure S6: Physiological state of the cultures from the co-cultivation experiment after four weeks of cultivation (T4). (A) Macroscopic visualization of cultures in MultiCultivator culture tubes. (B) Microscopic images of culture replicate R1. Scale bar is 10 µm.

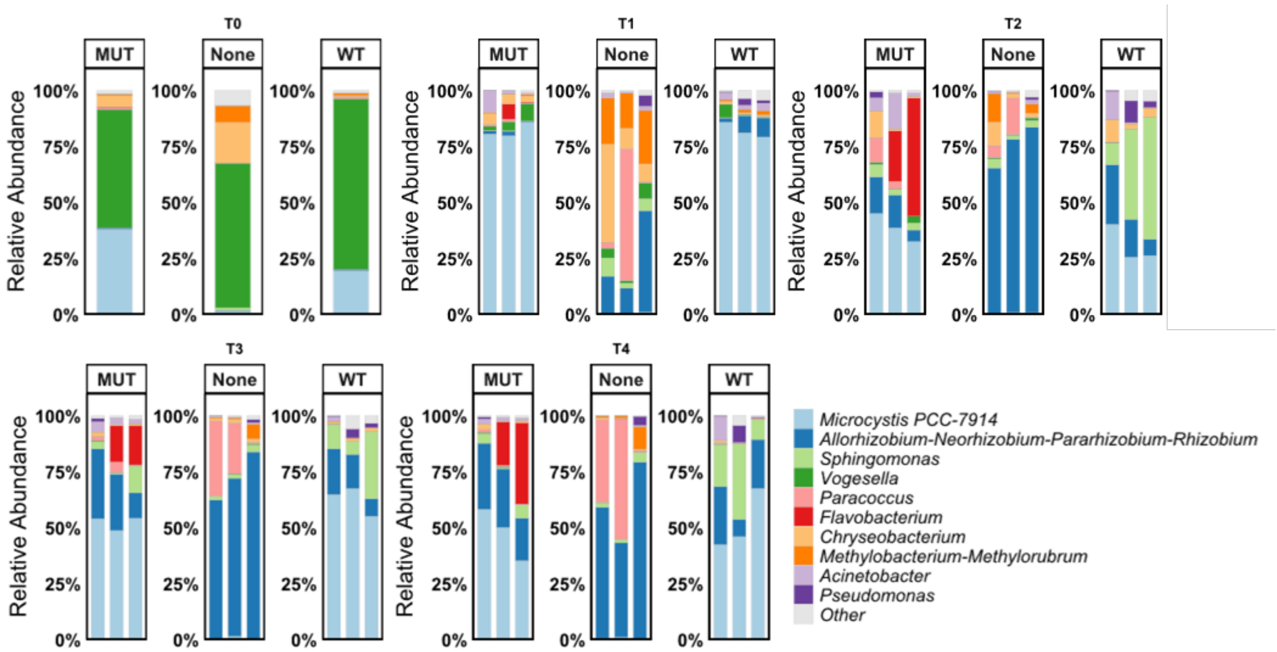

Figure S7: Relative abundances of heterotrophic bacterial genera in SynCom experiment. Each bar represents a replicate in the respective group. Sampling time points are indicated above the panels. MUT = co-cultivation with MC- strain, WT = co-cultivation with MC+ strain, None = cultivation of heterotrophic bacterial consortium without cyanobacteria.

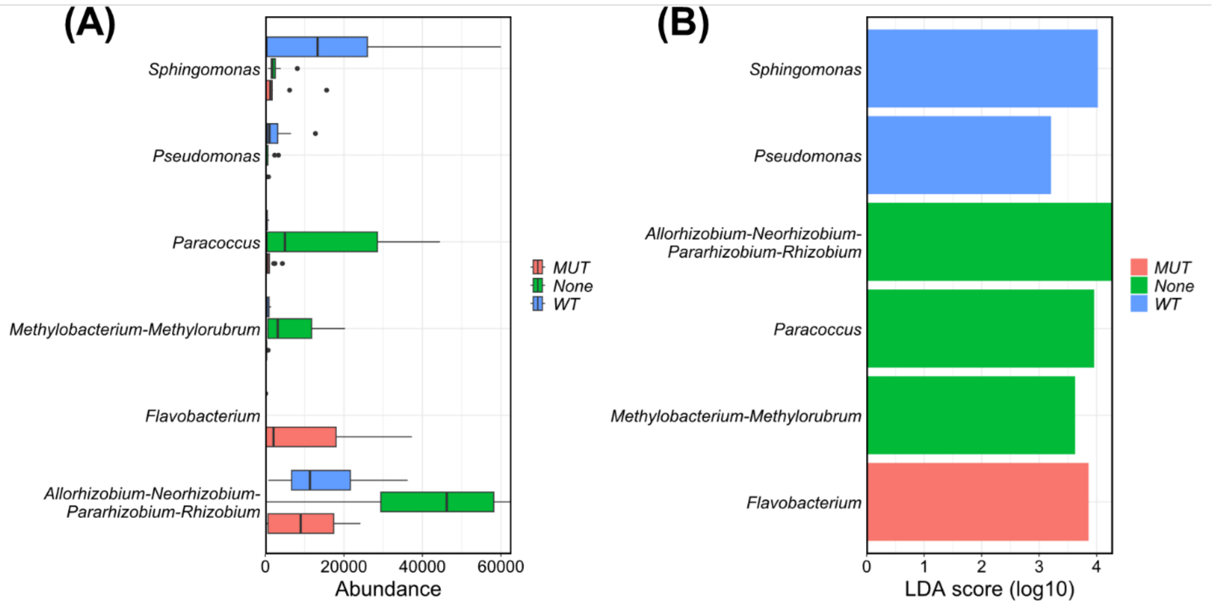

Figure S8: LEfSe analysis of SynCom experiment. (A) Boxplot of CSS abundance of taxa scored by LEfSe analysis of synthetic communities. (B) Log10-LEfSe Score of taxa with cutoff = 3.

Agrobacterium

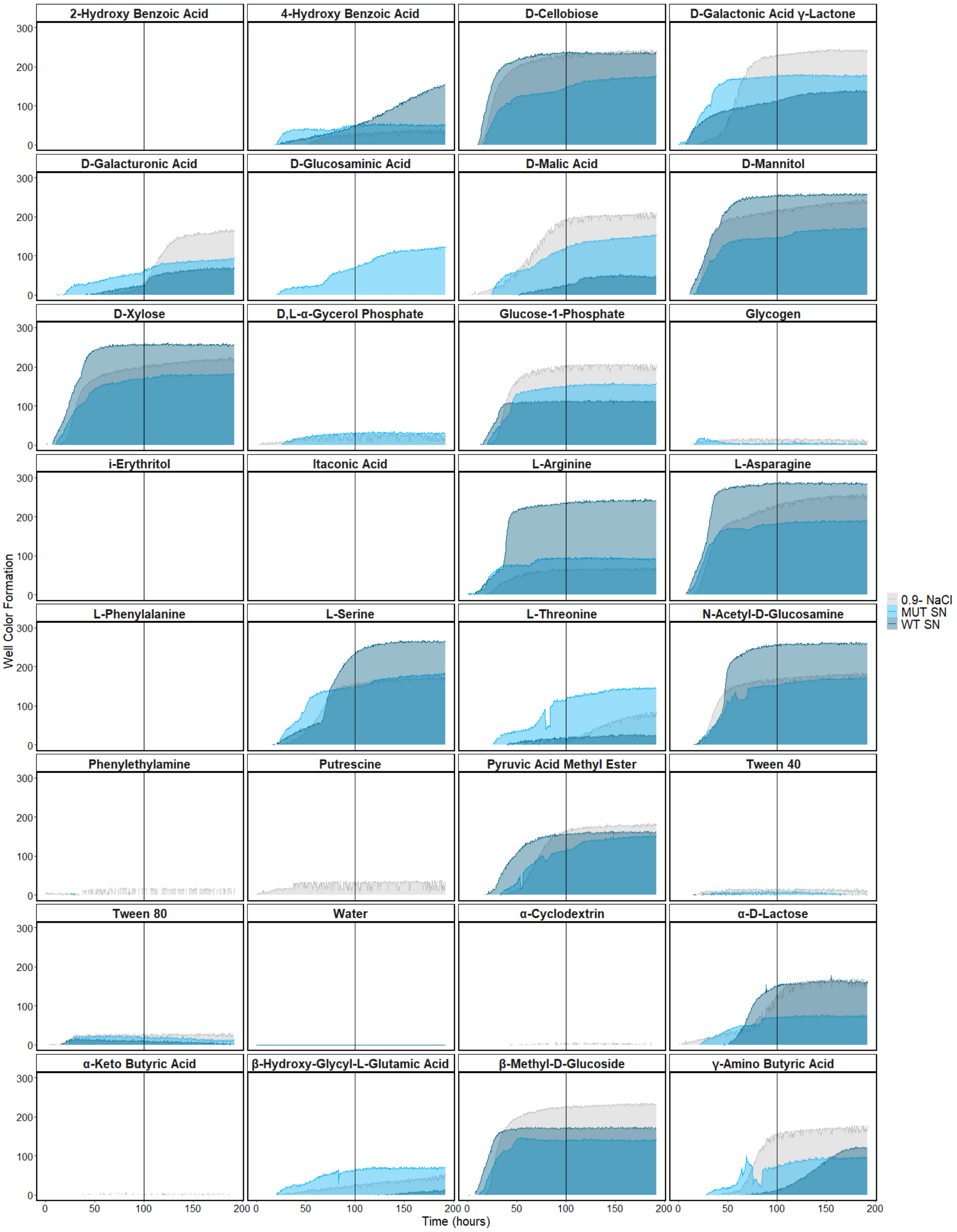

Figure S9: EcoPlate^{TM} substrate utilization test of *Agrobacterium* sp. UP1. Well color was measured as an indicator of metabolic activity by Biolog Software every 30 min for 192 hours. *Agrobacterium* was resuspended either in *Microcystis* culture exudates (WT exudates: dark blue, MUT (Δ*mcyB*) exudates: light blue) or 0,9%-NaCl solution (grey). Vertical line indicates the datapoint used for the heatmap (Fig. 4)

Sphingomonas

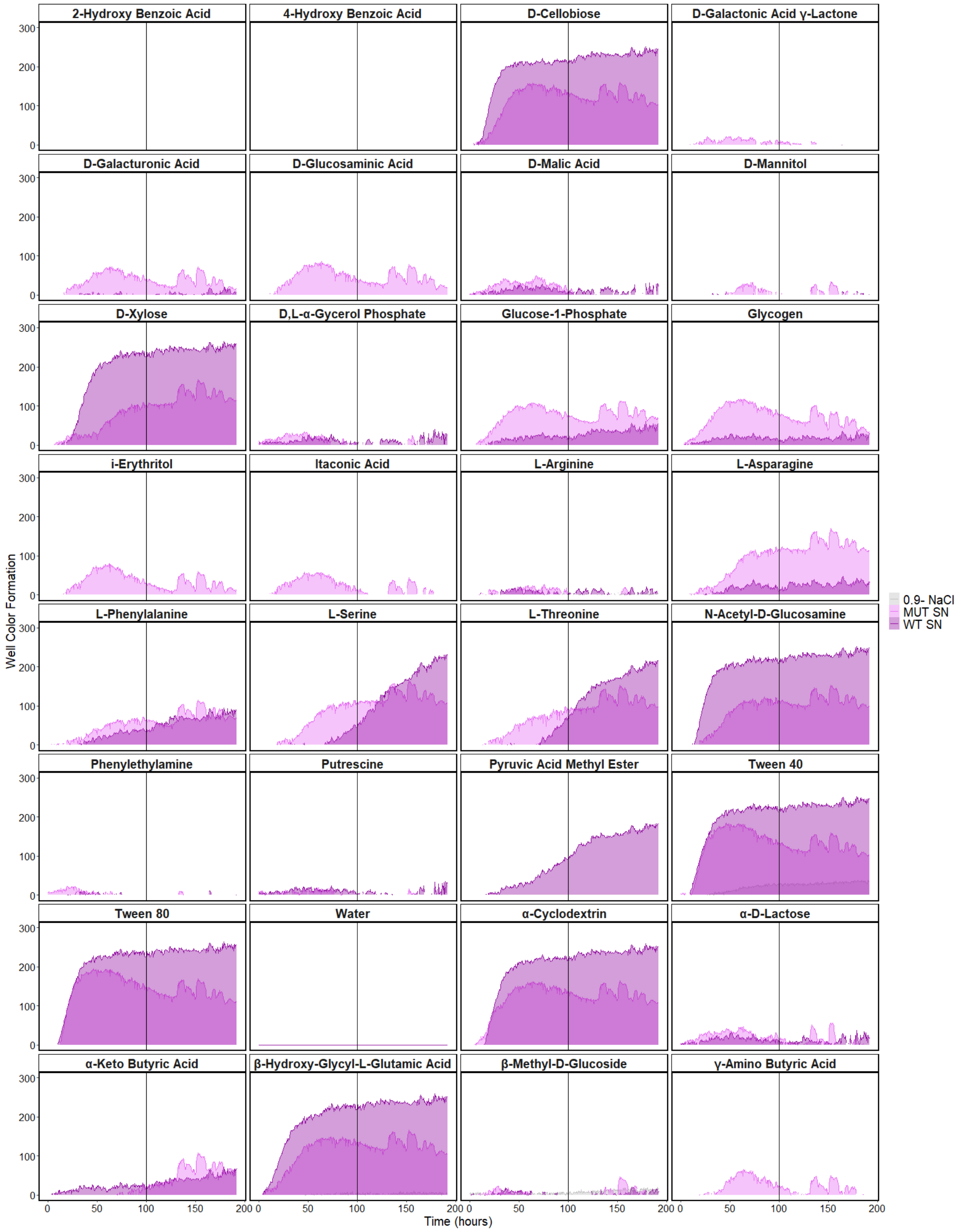

Figure S10: EcoPlate^{TM} substrate utilization test *Sphingomonas* sp. UP3. Well color was measured as an indicator of metabolic activity by Biolog Software every 30 min for 192 hours. *Sphingomonas* was resuspended either in Microcystis culture exudates (WT exudates: dark pink, MUT (Δ*mcyB*) exudates: light pink) or 0,9%-NaCl solution (grey). Vertical line indicates the datapoint used for the heatmap (Fig. 4)

Flavobacterium

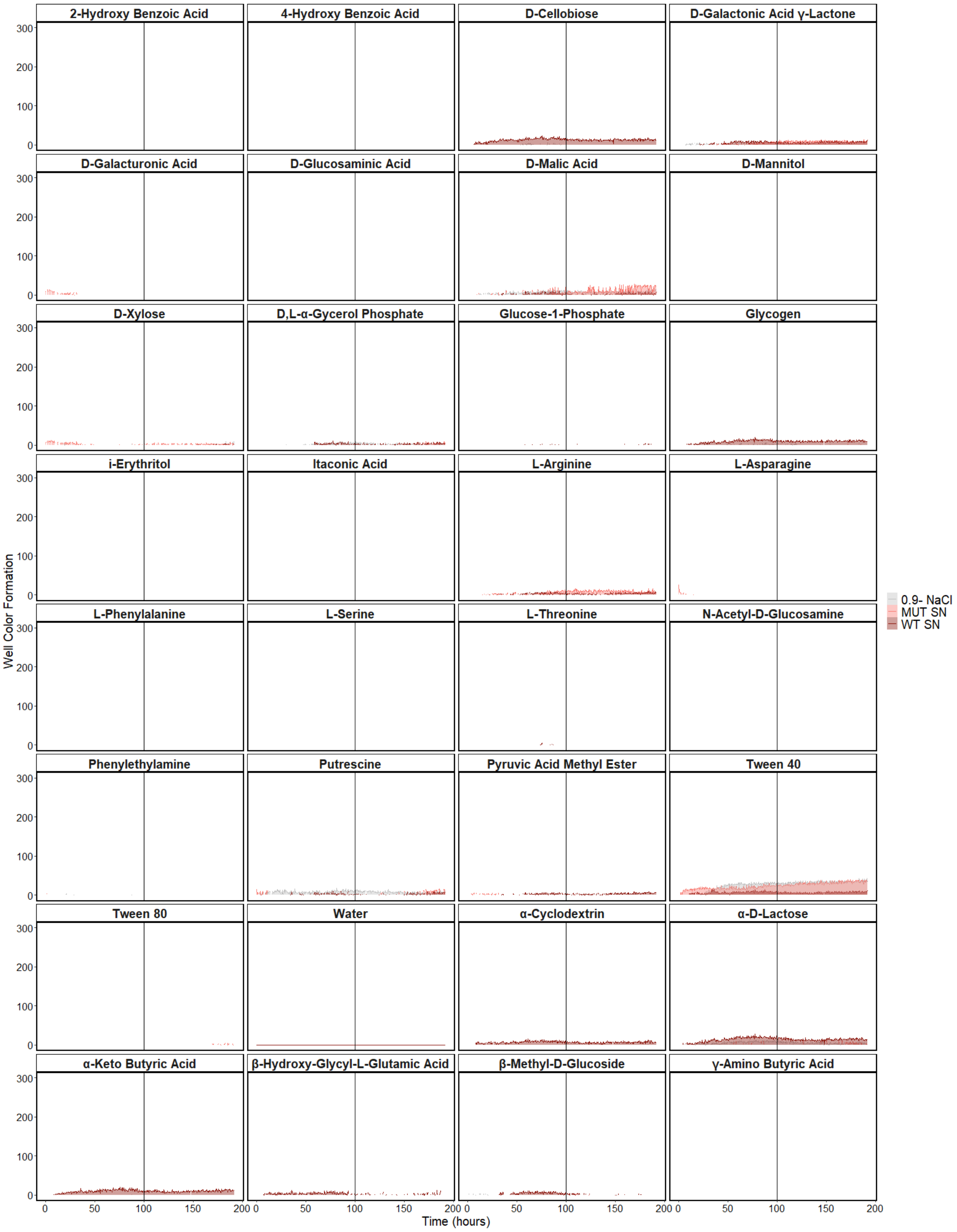

Figure S11: EcoPlate^{TM} substrate utilization test of *Flavobacterium* sp. UP2. Well color was measured as an indicator of metabolic activity by Biolog Software every 30 min for 192 hours. *Flavobacterium* was resuspended either in *Microcystis* culture exudates (WT exudates: dark pink, MUT (Δ*mcyB*) exudates: light pink) or 0,9%-NaCl solution (grey). Vertical line indicates the datapoint used for the heatmap (Fig. 4)

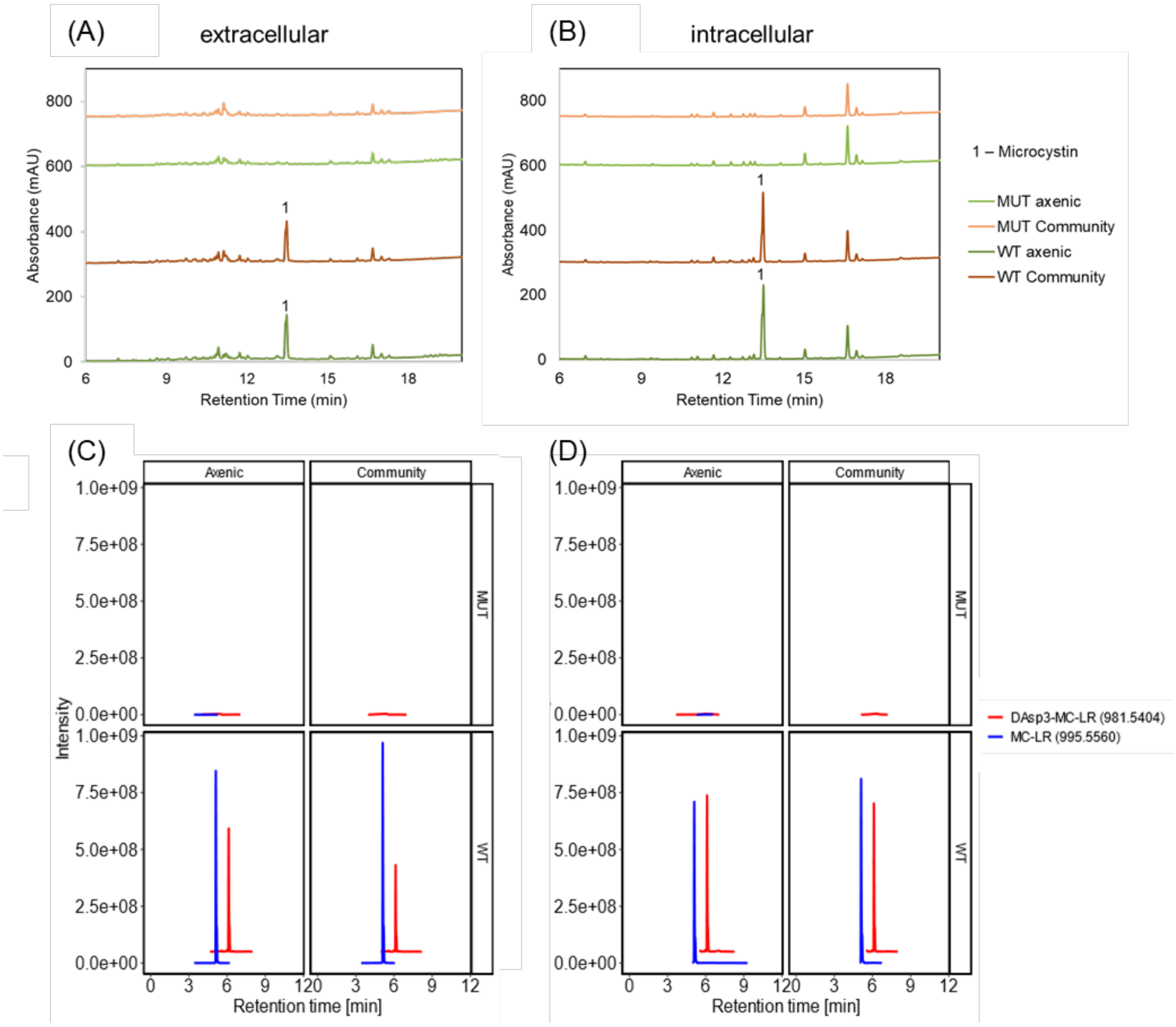

Figure S12: Comparative chromatograms of intracellular and extracellular extracts of *M. aeruginosa* PCC 7806 and Δ*mcyB* mutant monocultures (WT and MUT axenic) and cocultures additionally containing *Agrobacterium* sp. UP1, *Flavobacterium* sp. UP2 and *Sphingomonas* sp. UP3 (WT and MUT Community). Samples were taken after 14 days of cultivation from parallel grown mono- and cocultures. (A) and (B) show UV absorption for extra- and intracellular extracts, MC is indicated with **1**. (C) and (D) show extracted ion chromatograms (XICs) of Microcystin-LR (MC-LR) and its desmethyl variant (D-Asp3-MC-LR) from LC-MS measurements of the extracellular (C) and the intracellular (D) extract. Traces for D-Asp3-MC-LR in WT cultures are shifted both in Retention Time and Intensity to increase the plot readability.
